# Simultaneous coinfection with influenza virus and an arbovirus impedes influenza‐specific but not Semliki Forest virus–specific responses

**DOI:** 10.1111/imcb.70003

**Published:** 2025-02-19

**Authors:** Isabelle Jia‐Hui Foo, Aira F Cabug, Brad Gilbertson, John K Fazakerley, Katherine Kedzierska, Lukasz Kedzierski

**Affiliations:** ^1^ Department of Microbiology and Immunology The University of Melbourne, at the Peter Doherty Institute for Infection and Immunity Melbourne VIC Australia; ^2^ Department of Veterinary Biosciences, Faculty of Science University of Melbourne Melbourne VIC Australia

**Keywords:** CD8^+^ T cells, central nervous system, encephalitis, immunity to viral coinfections, influenza virus, Semliki Forest virus

## Abstract

Outbreaks of respiratory virus infections and arbovirus infections both pose a substantial threat to global public health. Clinically, both types of infection range from mild to severe and coinfections may occur more commonly than supposed. Our previous experimental coinfection study in mice demonstrated that prior infection with the arbovirus Semliki Forest virus (SFV) negatively impacted immune responses to influenza A virus (IAV). Here, we investigate whether simultaneous coinfection impacts the outcome of immune responses or disease. Simultaneous SFV and IAV infection did not lead to exacerbated or attenuated disease compared with the single virus infection control groups. SFV brain virus titers and brain pathology, including inflammation and immune responses, were comparable in the coinfection and single infection groups. By contrast, there was enhanced IAV replication, but no exacerbated lung pathology in coinfected mice. The magnitude of IAV‐specific CD8^+^ T‐cell responses in the lungs was lower compared with IAV‐only infection. Considered along with our previous study, this study provides evidence that the timing of viral coinfection is pivotal in determining effects on immune responses, pathological changes and disease outcome.

## Graphical abstract

In this study, we found that simultaneous infection with neurotropic Semliki Forest virus (SFV) and respiratory influenza A virus (IAV) does not lead to exacerbated or attenuated disease compared with the single virus infection control groups. However, coinfection impairs IAV immunity demonstrated by enhanced IAV replication in the lungs and decreased numbers of IAV‐specific CD8^+^ T cells in the lungs compared with IAV‐only infection. Conversely, IAV infection does not interfere with SFV immunity in simultaneous coinfection.

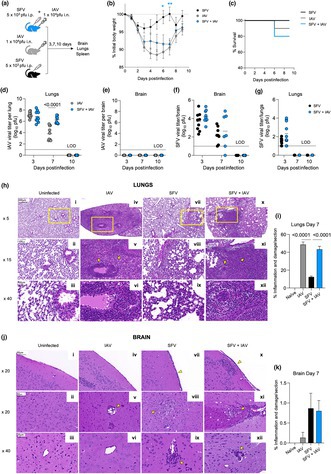

## INTRODUCTION

Coinfections of many kinds are commonly observed in real‐world human populations. Concomitant infections with two mosquito‐borne viruses can occur in settings where mosquito biting rates are high and there is a high abundance of mosquitoes infected with more than one arbovirus.^1^ Similarly, coinfection of viruses with different modes of transmission (e.g., respiratory and insect‐borne viruses) can also occur. Such situations are becoming increasingly common due to moderate to high levels of sustained severe acute respiratory syndrome coronavirus 2 (SARS‐CoV‐2) transmissions around the world, and unusual influenza seasonality patterns coinciding with mosquito population peaks in warmer months.^2^, ^3^ Understanding how simultaneous coinfections affect immune responses and disease outcomes provides insights for vaccine design and therapeutic applications.

Simultaneous coinfections may vary the temporal course and magnitude of immune responses. For example, specific immune responses are triggered by antigen‐presenting cells (APCs). A dual infection may reduce the number of APCs available to trigger immune responses for each virus, leading to both changes in the strength and quality of the immune response. This can affect the level of protection or contribute to immunopathology.^4^, ^5^ While many coinfection studies demonstrate that exposure to multiple pathogens concurrently is associated with greater disease severity, the consequences of simultaneous coinfections are not always predictable. Patients coinfected with influenza A virus (IAV) and human coronavirus have exacerbated disease severity compared with patients infected with IAV only.^6^ Conversely, patients coinfected with IAV and rhinovirus have lower disease severity.^6^ Vaccine interference, where one vaccine reduces antibody responses to another, have also been reported.^7^ Nigerian children who received smallpox, measles and yellow fever vaccines simultaneously at separate sites responded adequately to all three vaccines. However, when these same immunized children were given an additional diphtheria–pertussis–tetanus vaccine, there was a decrease in measles seroconversion rates.^8^

To investigate in more detail how concurrent exposure to two unrelated viruses affects immunity, we utilized our previously established mouse viral coinfection model using IAV and Semliki Forest virus (SFV).^9^ Here, we demonstrate that simultaneous coinfection hampers IAV‐specific, but not SFV‐specific immune responses by day 7 postinfection (dpi). Titers of infectious IAV and levels of lung inflammation were elevated in the simultaneously coinfected mice, with lower numbers of IAV‐specific CD8^+^ T cells in the lungs. However, both IAV‐ and SFV‐specific long‐term memory CD8^+^ T‐cell pools were comparable in magnitude across infection type.

## RESULTS

### Simultaneous coinfection with SFV + IAV leads to higher lung IAV viral titer

**Figure 1:**
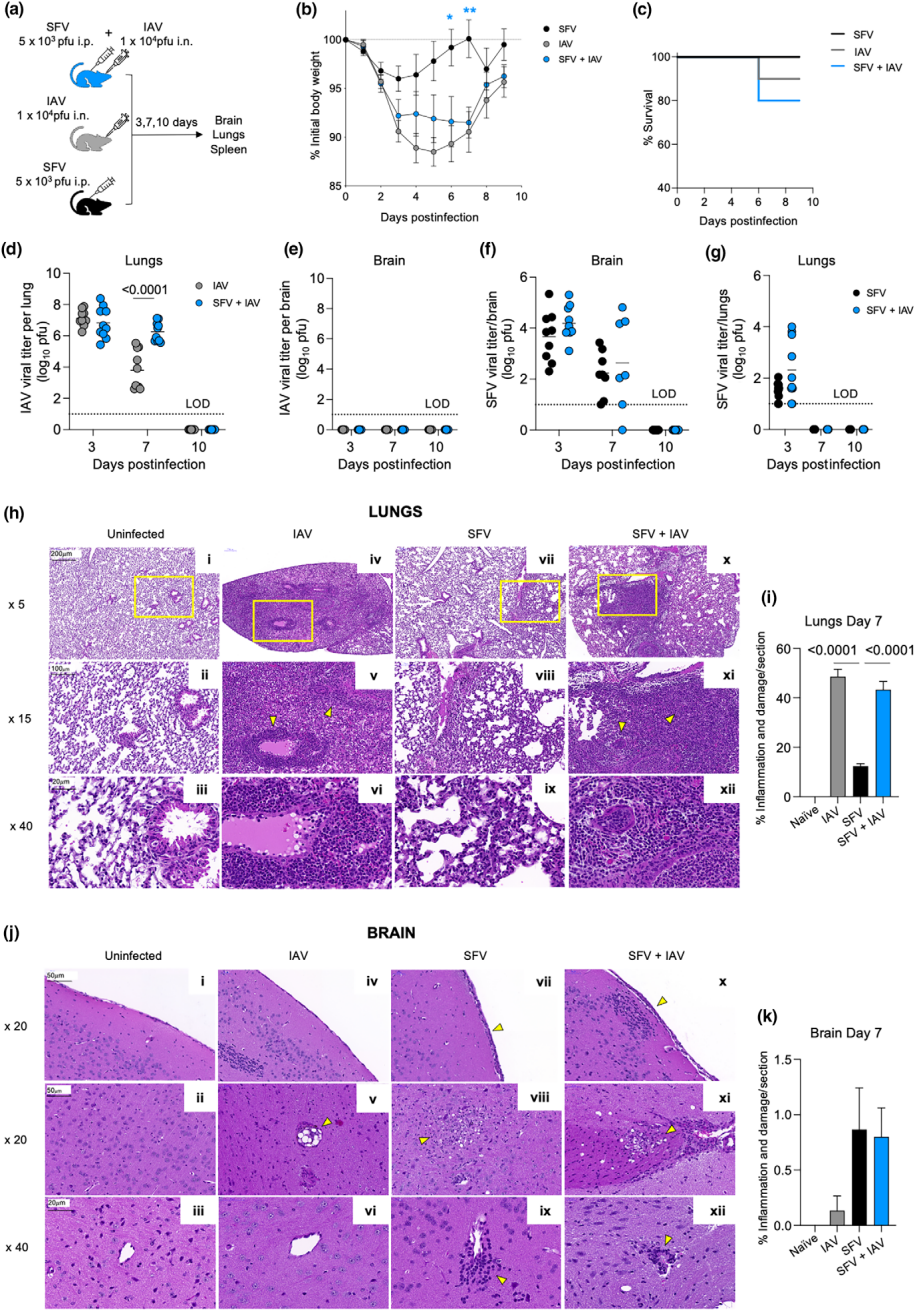
Simultaneous coinfection of Semliki Forest virus (SFV) + influenza A virus (IAV) leads to higher lung IAV virus titers. **(a)** Mice were infected at the same time with 10^4^ plaque‐forming units (pfu) A/HKx31 [intranasally (i.n.)] and 5 × 10^3^ pfu A7(74) SFV [intraperitoneally (i.p.)]. The SFV‐only and IAV‐only groups were included as controls. Brain, lungs and spleens were analyzed at 3, 7 and 10 days postinfection (dpi). **(b)** Weight loss and **(c)** survival of SFV + IAV–, IAV‐ and SFV‐infected mice monitored for 10 days (*n* = 10, two independent experiments, error bars represent standard deviation). IAV infectivity titers in the **(d)** lungs and **(e)** brain were determined by plaque assay on Madin–Darby canine kidney (MDCK) cells. Each symbol denotes an individual mouse (*n* = 10, two independent experiments, error bars represent mean). Significance was determined by the Student's unpaired *t*‐test. SFV infectivity titers in the **(f)** brain and **(g)** lungs were determined by a plaque assay on Vero cells. Each symbol denotes an individual mouse (*n* = 7–10, two independent experiments, error bars represent mean). Histopathological changes in the **(h)** lungs and **(j)** brains of SFV‐, IAV‐ and SFV + IAV–infected mice on 7 dpi. Brains and lungs from uninfected mice are included as controls. Tissue sections were stained with hematoxylin and eosin; representative images are shown. Bar = 200 μm for 5×; 100 μm for 15×; 50 μm for 20× and 20 μm for 40× magnification. Yellow arrowheads point to areas of inflammation and damage as described in the text, and 15× images are the magnified areas outlined in yellow at 5× magnification. Quantification of the extent of inflammation and damage in the SFV, IAV and SFV + IAV groups in the **(i)** lungs and **(k)** brains (*n* = 3, error bars represent standard error of the mean). Significance was determined by Tukey's multiple comparison test. LOD, limit of detection. *P < 0.05, **P < 0.005.

Adult C57BL/6J mice were infected with either 5 × 10^3^ plaque‐forming units (pfu) A7(74) SFV via the intraperitoneal route, 10^4^ pfu A/HKx31 (x31) IAV via the intranasal route or with both viruses at the same time (SFV + IAV simultaneous infection) (Figure 1a). In mice, the A7(74) strain is 100% neuroinvasive following intraperitoneal injection and does not require intracranial inoculation. The dose used is routinely used in our studies leading to a weight loss of up to 10% of initial body weight, and robust immune responses in the brain. Similarly, IAV (x31; H3N2) has been routinely used in our laboratory to elicit robust immune responses, but not to kill animals or lead to excessive body weight loss. IAV x31 is given intranasally in our study to mimic a natural route of infection, while SFV A7(74) is given intraperitoneally instead, as subcutaneous inoculation of SFV is not commonly used. Experimentally, SFV can infect mice by many routes of inoculation, including intraperitoneal, intranasal, intracranial, intravenous, subcutaneous or intramuscular.^10^, ^11^, ^12^ In each case, SFV is readily neuroinvasive and is detectable in the brain. As intraperitoneal injection is the most widely used method of SFV inoculation, we have not performed other modes of infection.

Weight loss following coinfection was recorded over 10 days. Mice were killed in accordance with the ethics requirements, whereby coinfected mice with ≥ 20% body weight loss must be humanly killed. Mice in the SFV + IAV group had lost approximately 10% of their initial body weight by 3–7 dpi (Figure 1b, c). Compared with IAV‐infected mice, body weight loss of SFV + IAV mice was similar between both groups, implying that body weight loss observed in SFV + IAV mice was mainly driven by the IAV infection^13^. Conversely, SFV + IAV–coinfected mice had a significantly greater body weight loss than SFV‐only infected controls (Figure 1c). SFV‐only infection with the A7(74) strain generally does not cause body weight loss of more than 5–10% and this was confirmed by our data.^14^, ^15^ However, we found that 20% (2/10 mice) of coinfected mice died due to the disease (20% body weight loss), despite the majority of animals showing mild body weight loss compared with IAV‐infected mice (Figure 1d). By contrast, IAV‐only control had 90% survival rate (9/10 mice), and all SFV‐only infected mice survived (10/10 mice; Figure 1d).

To determine infectious virus titers in single *versus* coinfected mice, brains and lungs were collected at 3, 7 and 10 dpi. Simultaneous coinfection with SFV and IAV led to significantly higher titers of infectious IAV in the lungs of coinfected mice at 7 dpi (Figure 1e). This phenomenon was also observed in our previous study, where sequentially infected mice (SFV → IAV) had lower lung IAV viral titers at 3 dpi, but significantly higher lung viral titers by 7 dpi compared with IAV‐only control.^9^ Infectious IAV was not detectable at 10 dpi in the lungs (Figure 1e), and no infectious IAV was isolated from the brains of IAV‐only and SFV + IAV–infected mice (Figure 1f). Conversely, we found that both SFV‐only and SFV + IAV mice had comparable SFV viral titers in the brain at 3 and 7 dpi, while no virus was detected at 10 dpi (Figure 1f). Similarly, in the lungs, SFV viral titers were comparable in SFV and coinfected mice at 3 dpi (Figure 1g). It is not entirely unexpected to detect SFV in lungs on day 3 postinfection, as SFV‐based reporter vectors have been detected in the lungs, heart, spleen and kidneys upon systemic infection via intravenous or intraperitoneal routes.^11^ The SFV presence in the lungs is most likely due to the blood traversing through lungs, and on day 3 there is still a high blood viremia as previously shown.^9^, ^16^ Our previous work^17^ demonstrated that type I interferon (IFN) system effectively curtails the spread of SFV in different tissues, limiting its infectivity. However, in the absence of type I IFN, the virus enters and replicates in all permissive cell types, including the alveolar lining cells.

### Simultaneous coinfection does not lead to exacerbated pathology in the brain and lungs

To evaluate the histopathological changes in SFV (*n* = 3), IAV (*n* = 3) and SFV + IAV (*n* = 3) mice, hematoxylin and eosin–stained sections from the lungs and brains were assessed and scored on 7 dpi. We demonstrated that the lungs of both SFV + IAV– and IAV‐infected mice had comparable levels of histopathological changes typical for the IAV infection (Figure 1h, i), and both groups displayed significantly higher percentage of inflammation and damage compared with the lungs of SFV‐only infection (Figure 1i). During coinfection or IAV‐only infection, lungs displayed multifocal interstitial and intra‐alveolar mixed cell infiltration consisting of both mononuclear and polymorphonuclear cells, vasculitis, perivasculitis, bronchiolitis and hemorrhage (Figure 1h, panels v, vi, xi, xii). By contrast, SFV‐infected mice showed mild inflammation and damage exemplified by mild focal intra‐alveolar lymphocytic infiltration and septal thickening as a result of macrophage infiltrations (Figure 1h, panels viii and ix). Despite higher IAV viral load in the lungs following coinfection, exacerbated lung pathology was not observed in the lungs of coinfected animals (Figure 1i).

In the brains of SFV‐only and SFV + IAV mice, changes consisted of meningeal infiltration (Figure 1j, panels vii and x), neuronal necrosis and microgliosis (Figure 1j, panels viii and xi) and perivascular cuffing (Figure 1j, panels ix and xii), all characteristic of SFV infection. By contrast, brains of IAV‐infected mice displayed minimal to no inflammation or damage, except for focal vacuolation and associated mononuclear cell infiltration observed in the midbrain of one mouse (Figure 1j panel v). Overall, SFV and SFV + IAV mice had histopathological changes that were similar in both nature and extent (Figure 1k).

### Increased cytokine and chemokine levels in the lungs at 7 dpi in SFV + IAV mice

**Figure 2:**
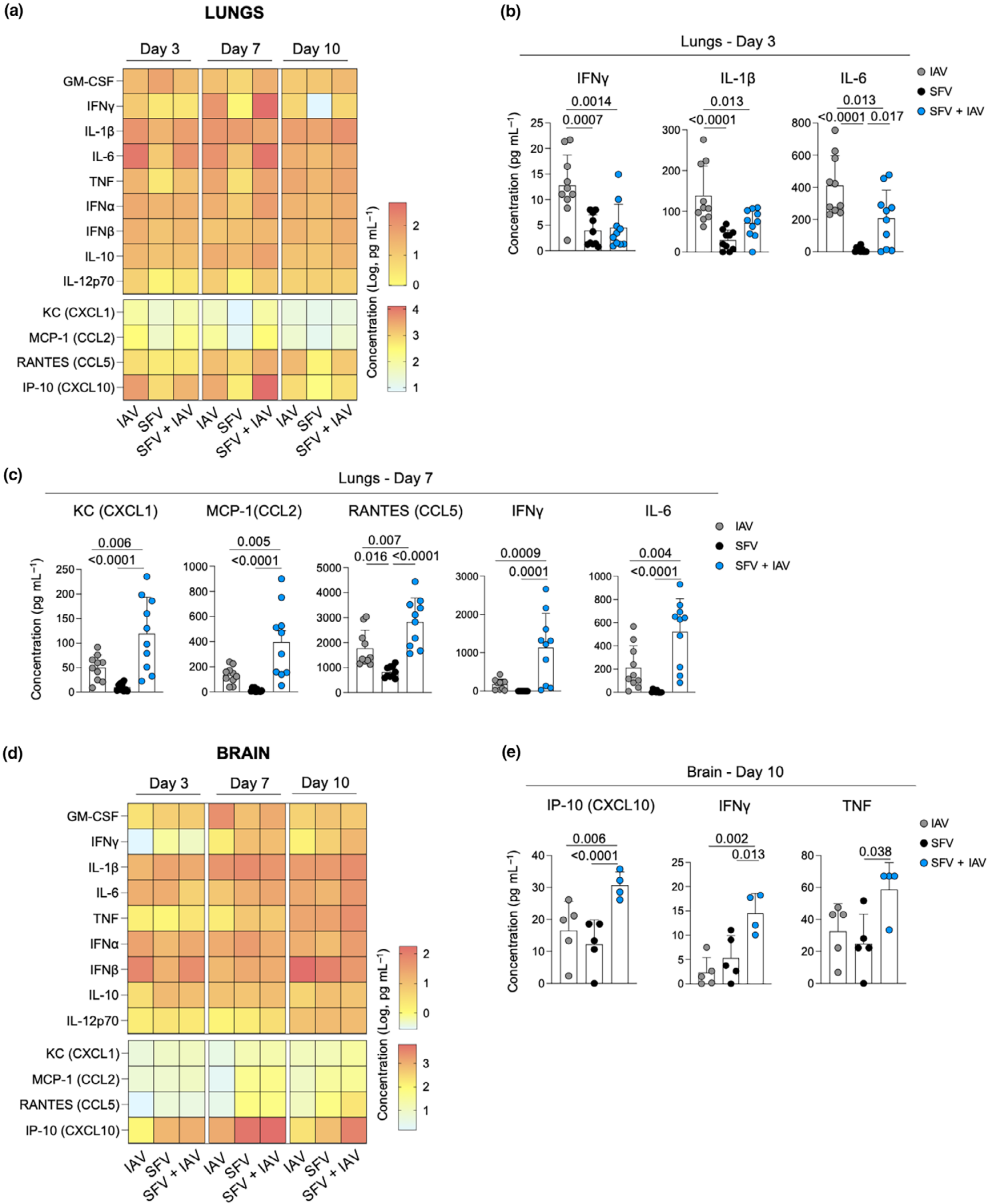
Cytokine and chemokine profiles in the brains and lungs of Semliki Forest virus (SFV)–, influenza A virus (IAV)– and SFV + IAV–infected mice. **(a)** Heatmaps summarizing the differences in cytokine and chemokine concentrations in lung homogenates across time points for each infection type (*n* = 10 in each group and time point, two independent experiments). **(b, c)** Comparison of significantly different chemokines in the lungs (*n* = 10 per time point, two independent experiments, error bars represent standard deviation). **(d)** Heatmaps summarizing the differences in cytokine and chemokine concentrations in brain homogenates across time points in each infection type (*n* = 10 in each group and time point, two independent experiments). **(e)** Comparison of significantly different cytokines and chemokines in the brain (*n* = 5–10 per time point, two independent experiments, error bars represent standard deviation). Each symbol denotes an individual mouse. Significance was determined by Tukey's multiple comparison test. CCL, C–C motif chemokine ligand; CXCL, C–X–C motif chemokine ligand 1; GM‐CSF, granulocyte–macrophage colony‐stimulating factor; IFN, interferon; IL, interleukin; IP‐10, interferon gamma–induced protein 10; KC, keratinocyte‐derived chemokine; MCP‐1, monocyte chemoattractant protein‐1; RANTES, regulated upon activation, normal T‐cell expressed and secreted; TNF, tumor necrosis factor.

To characterize the inflammation elicited by SFV + IAV coinfection, compared with IAV‐ and SFV‐single infections, we assessed the production of 13 different cytokines and chemokines in lung and brain homogenates at 3, 7 and 10 dpi (Figure 2a, d). At 3 dpi, levels of IFNγ, interleukin‐1β and interleukin‐6 were significantly higher in the lungs of IAV‐infected mice compared with SFV‐ or SFV + IAV–infected mice (Figure 2b). By 7 dpi, the SFV + IAV coinfection had induced significantly higher levels of chemokines keratinocyte‐derived chemokine [KC; also known as C–X–C motif chemokine ligand 1 (CXCL1)], monocyte chemoattractant protein‐1 (MCP‐1; also known as CCL2) and regulated upon activation, normal T‐cell expressed and secreted (RANTES; also known as CCL5) in the lungs compared with IAV‐ and SFV‐only infection and levels of IFNγ and interleukin‐6 were now higher in the coinfected mice than in the IAV‐infected mice, a switch relative to day 3 (Figure 2c). Elevated levels of proinflammatory mediators MCP‐1, IFNγ and interleukin‐6 have previously been associated with severe and fatal influenza disease in humans as well as in our previous sequential coinfection study.^9^, ^18^

In the brain, there were significantly higher levels of interferon gamma–induced protein 10 (IP‐10 or CXCL10), IFNγ and tumor necrosis factor in the SFV + IAV–coinfected mice compared with SFV‐ and IAV‐only infections at 10 dpi (Figure 2d, e). Otherwise, the overall cytokine and chemokine levels in the brain between infection types were comparable across time points.

### Coinfection changes the magnitude of cellular immune responses in virus‐infected tissues

**Figure 3:**
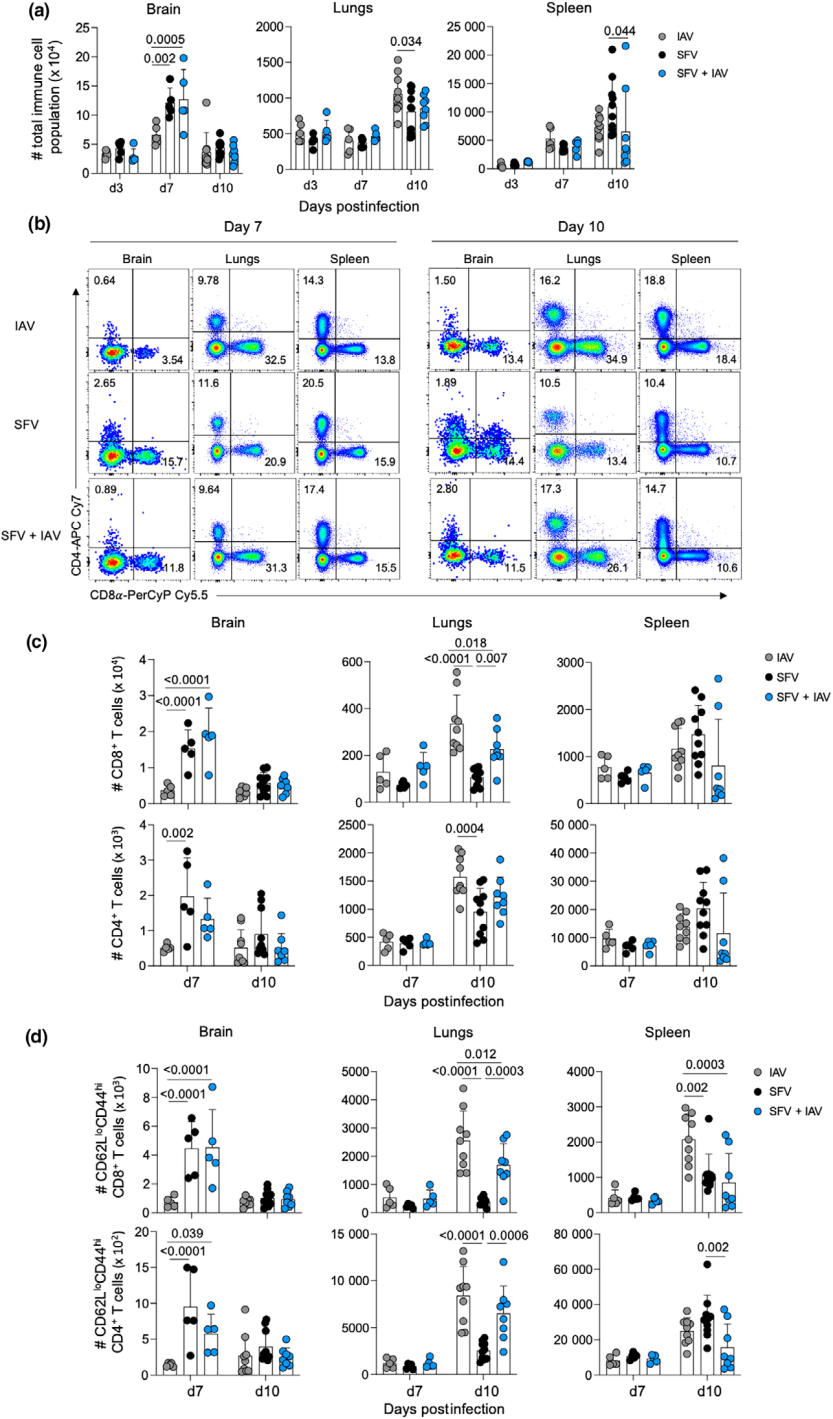
Analysis of total immune cell numbers in Semliki Forest virus (SFV)–, influenza A virus (IAV)– and SFV + IAV–infected mice at 7 and 10 days postinfection (dpi). **(a)** Absolute numbers of leukocytes in the brain, lungs and spleen of IAV‐, SFV‐ and SFV + IAV–infected mice at 3, 7 and 10 dpi; (*n* = 5–10, two independent experiments, error bars represent standard deviation). **(b)** Representative fluorescence‐activated cell sorting (FACS) plots show the proportion of CD8^+^ and CD4^+^ T cells across different tissues in SFV‐, IAV‐ and SFV + IAV–infected mice at 7 and 10 dpi. **(c)** Absolute numbers of CD8^+^ (top row) and CD4^+^ (bottom row) T cells across different anatomical sites in SFV‐, IAV‐ and SFV + IAV–infected mice at 7 and 10 dpi (*n* = 5–10, two independent experiments error bars represent standard deviation). **(d)** Absolute numbers of activated (CD44^+^ CD62L^lo^) CD8^+^ (top row) and CD4^+^ (bottom row) T cells across different anatomical sites in IAV‐, SFV‐ and SFV + IAV–infected mice at 7 and 10 dpi (*n* = 5–10, two independent experiments, error bars represent standard deviation). Each symbol denotes an individual mouse. Significance was determined by Tukey's multiple comparison test.

While the lungs and brains exhibited similar pathology across the infection groups, we demonstrated changes in the viral load and cytokine levels in different groups. Therefore, we conducted an in‐depth investigation to determine whether coinfection caused any changes or perturbations in the immune responses. To do this, we performed a broad analysis of myeloid cells (Supplementary figure 1a–e), B cells (Supplementary figure 2a, b) and T cells (Figure 3b, c) in the brain, lungs and spleens at 3 dpi (myeloid compartment only), and at 7 and 10 dpi. In the brain, both SFV‐only and SFV + IAV had higher numbers of cellular infiltrates compared with IAV‐only infection at 7 dpi (Figure 3a). In the lungs, IAV infection induced greater cellular infiltration at 10 dpi compared with SFV‐only infection (Figure 3a). In the spleen, SFV‐only infection led to a greater number of cellular infiltrates at 10 dpi compared with that of coinfection (Figure 3a).

Innate immune responses in the brain in SFV + IAV coinfection led to increased numbers of inflammatory monocytes and dendritic cells compared with the SFV‐only group, while differences in macrophages were only observed between the IAV‐only and the other two groups (Supplementary figure 1b–d). The numbers of lung inflammatory monocytes were higher in SFV + IAV compared with IAV‐only infection, while the number of dendritic cells and alveolar macrophages in the lungs were higher in the IAV‐only infection group (Supplementary figure 1c–e). Interestingly, at 7 dpi we found increased B‐cell infiltration in the brain of IAV‐only infection in comparison to the SFV‐only and SFV + IAV infection groups (Supplementary figure 1f). In the spleen, the SFV‐only infection had greater B‐cell responses compared with both IAV and SFV + IAV. B‐cell numbers were comparable between groups in the lungs (Supplementary figure 1f, g).

Comprehensive analyses of T‐cell responses showed that SFV + IAV– and SFV‐infected mice had greater brain CD8^+^ T‐cell infiltration compared with IAV infection at 7 dpi, while SFV‐only infection had more CD4^+^ T cells in the brain compared with IAV infection (Figure 3b, c). Conversely, IAV infection caused a higher influx of CD4^+^ and CD8^+^ T cells in the lungs at 10 dpi compared with SFV‐ or coinfection. There were no differences in CD4^+^ and CD8^+^ T‐cell numbers across infection groups and time points in the spleen (Figure 3b, c).

Effector CD4^+^ and CD8^+^ T‐cell numbers in the brains of SFV‐ and SFV + IAV–infected mice were higher than in the IAV‐only group at 7 dpi (Figure 3d). In the lungs, higher numbers of effector CD4^+^ and CD8^+^ T cells were observed in both IAV and coinfection compared with the SFV‐only group at 10 dpi (Figure 3d). In contrast to the total CD4^+^ and CD8^+^ T‐cell findings in the spleen, IAV‐only infection significantly increased the infiltration of the number of effector CD8^+^ T cells in the spleen compared with both SFV and coinfection at 10 dpi. However, SFV‐only infection had significantly higher effector CD4^+^ T‐cell infiltration compared with coinfection at 10 dpi in the spleen with no differences observed between the IAV‐ and SFV‐only groups (Figure 3d).

**Figure 4:**
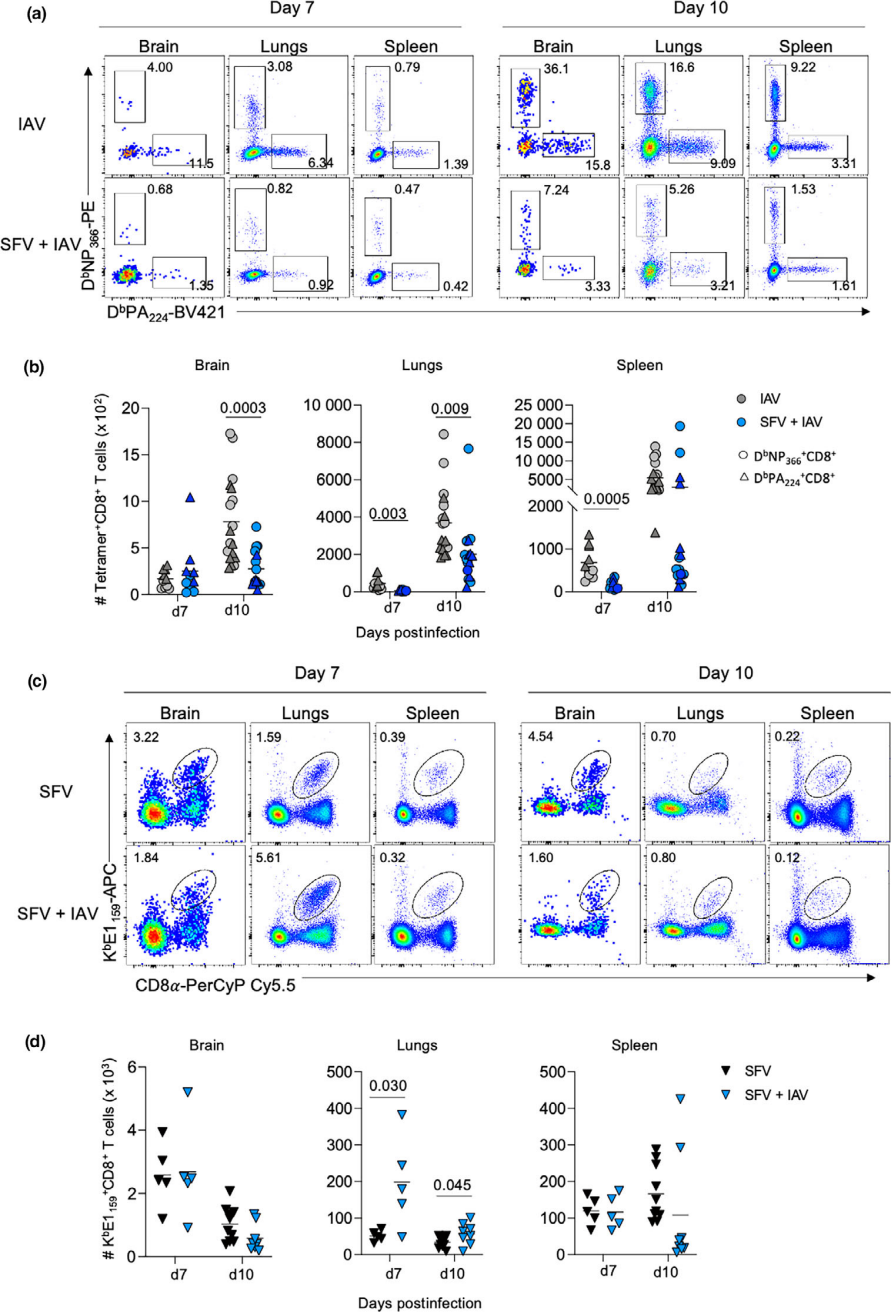
Analysis of virus‐specific CD8^+^ T cells across different anatomical sites in each infection type at 7 and 10 days postinfection (dpi). **(a)** Representative fluorescence‐activated cell sorting (FACS) plots show the proportion of influenza A virus (IAV)–specific CD8^+^ T cells of D^b^NP_366_ and D^b^PA_224_ specificities across different tissues in IAV‐ and Semliki Forest virus (SFV) + IAV–infected mice at 7 and 10 dpi. **(b)** Absolute numbers of IAV‐specific CD8^+^ T cells of D^b^NP_366_ and D^b^PA_224_ specificities across different anatomical sites in IAV‐ and SFV + IAV–infected mice at 7 and 10 dpi. (*n* = 5–10, error bars represent mean). **(c)** Representative FACS plots show the proportion of SFV‐specific CD8^+^ T cells directed at K^b^E1_159_ epitope across different tissues of SFV‐ and SFV + IAV–infected mice at 7 and 10 dpi. **(d)** Absolute numbers of SFV‐specific CD8^+^ T cells directed at K^b^E1_159_ epitope across different anatomical sites of SFV‐ and SFV + IAV–infected mice at 7 and 10 dpi (*n* = 5–10, two independent experiments, error bars represent mean). Each symbol denotes an individual mouse. Significance was determined by the Student's unpaired *t*‐test.

To investigate immune responses toward IAV and SFV at the epitope level, we utilized IAV‐ and SFV‐specific major histocompatibility complex (MHC)‐I tetramers (D^b^NP_366_ and D^b^PA_224_; two immunodominant CD8^+^ T‐cell IAV epitopes in B6 mice^19^ and K^b^E1_159‐166_; an immunodominant CD8^+^ T‐cell SFV epitope in B6 mice^9^) to identify virus‐specific (either IAV or SFV) effector CD8^+^ T cells. IAV‐specific responses were greater in IAV‐only infection in the brain, lungs and spleen compared with coinfection (Figure 4a, b). Conversely, SFV + IAV coinfection led to significantly increased numbers of SFV‐specific CD8^+^ T cells in the lungs compared with SFV‐only infection at 7 and 10 dpi. No differences in CD8^+^ T‐cell responses between SFV and SFV + IAV infection in the brain and spleen were observed across time points (Figure 4c, d). This result agrees with our findings regarding inflammatory mediators, where overall higher cytokine and chemokine levels in the lungs of coinfected mice were potentially responsible for recruiting more lymphocytes to the site of infection compared with that of SFV‐only infection (Figure 2c).

### Coinfection modulates the activation profile of T‐cell populations in target tissues

**Figure 5:**
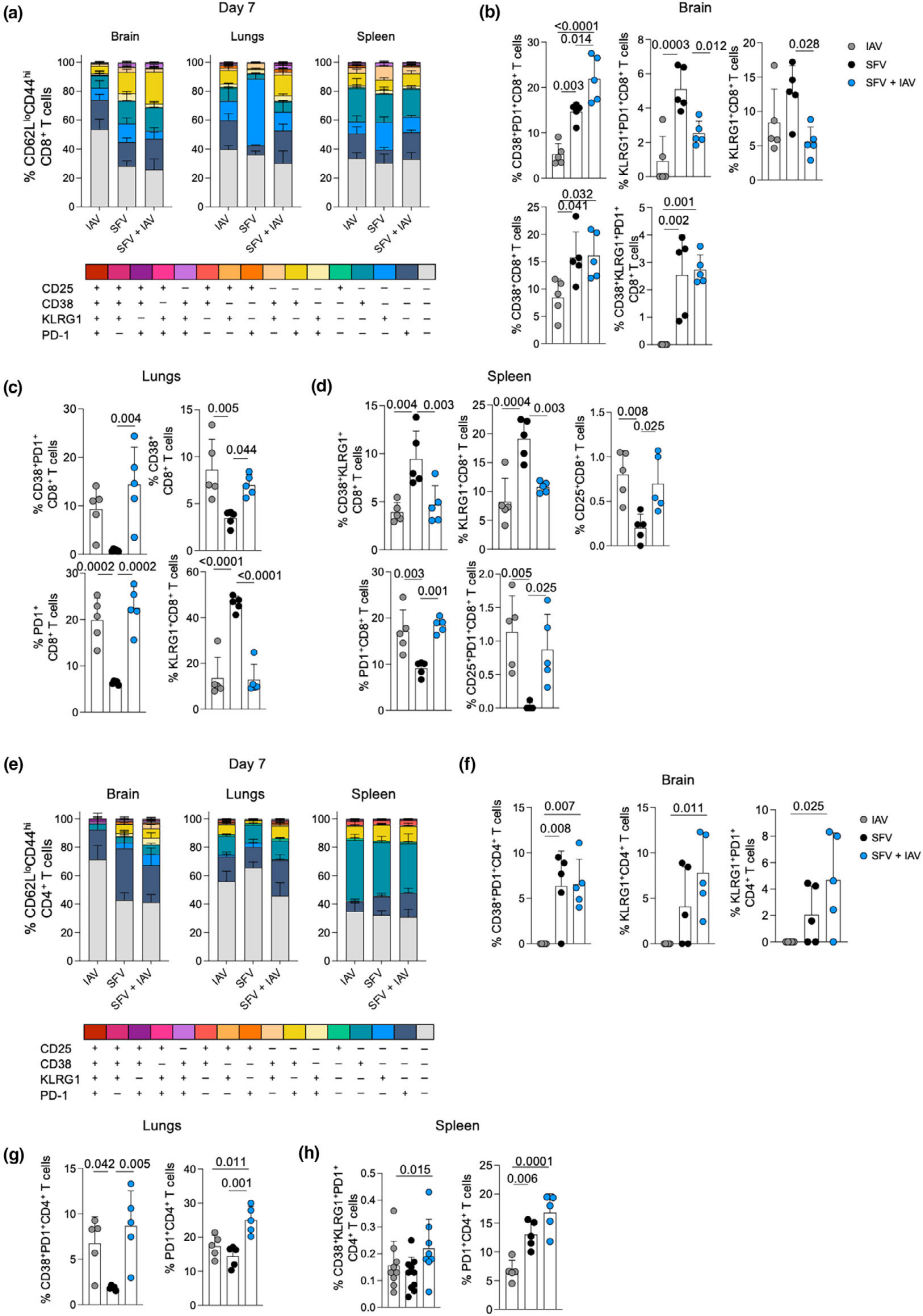
Activation profiles of effector CD4^+^ T cells and CD8^+^ T cells across anatomical sites in influenza A virus (IAV)–, Semliki Forest virus (SFV)– and SFV + IAV–infected mice at 7 days postinfection (dpi). Stacked bar graphs depicting frequencies of activation marker combinations on **(a)** CD8^+^ T cells and **(e)** CD4^+^ T cells on 7 dpi in IAV‐, SFV‐ and SFV + IAV–infected mice; (*n* = 5, error bar represents standard deviation). Comparison of significantly different activation marker combinations in the brain, lungs and spleen on CD8^+^ T cells **(b–d)** and CD4^+^ T cells **(f–h)** on 7 dpi (*n* = 5, error bar represents standard deviation). Each symbol denotes an individual mouse. Significance was determined by Tukey's multiple comparisons test. PD‐1, programmed cell death protein 1.

To further characterize effector CD4^+^and CD8^+^ T‐cell responses, we examined key T‐cell activation markers [CD25, CD38, KLRG1 and programmed cell death protein 1 (PD‐1)] in the brain, lungs and spleen of SFV‐, IAV‐ and SFV + IAV–infected mice. In the brain, SFV‐only infected mice had significantly higher frequencies of effector CD8^+^ T cells expressing KLRG1, CD38 and PD‐1 compared with IAV and SFV + IAV infection, whereas in SFV + IAV infection, CD38 and PD‐1 were coexpressed at higher frequency (Figure 5a, b and Supplementary figure 3b). Conversely, lungs of IAV‐only–infected mice displayed significantly higher frequencies of effector CD8^+^ T cells expressing either CD38 or PD‐1, whereas the CD8^+^ T cells in coinfection had a CD38^+^ PD‐1^+^ phenotype. KLRG1 was highly expressed on CD8^+^ T cells in SFV‐infected lungs (Figure 5c; Supplementary figure 3c). In the spleen, effector CD8^+^ T cells in SFV infection displayed increased expression of KLRG1^+^ and CD38^+^ KLRG1^+^ phenotypes; and PD‐1^+^ and CD38^+^ phenotypes in IAV infection (Figure 5d; Supplementary figure 3d).

Effector CD4^+^ T cells in the brains of the SFV‐only and SFV + IAV–coinfected groups displayed similar activation phenotype of CD38^+^ PD1^+^, KLRG1^+^ compared with IAV‐only infection (Figure 5e, f). In addition, CD4^+^ T cells of IAV‐only infection in the brain had a higher frequency of single‐positive CD38^+^ cells than the SFV‐only and coinfection groups by 10 dpi (Supplementary figure 2f). Conversely, the effector CD4^+^ T‐cell population in IAV‐ and SFV + IAV–infected lungs displayed increased levels of both single‐positive and double‐positive CD38^+^ PD1^+^ phenotype cells compared with SFV infection at 7 and 10 dpi (Figure 5g and Supplementary figure 3g). The KLRG1^+^ phenotype was again observed in the lungs CD4^+^ T cells of SFV‐infected mice at 10 dpi, similar to our observation in CD8^+^ T cells (Supplementary figure 2c, g). Finally, CD38^+^ KLRG1^+^ PD1^+^ triple‐positive and single‐positive PD1^+^ phenotype was observed in spleen effector CD4^+^ T cells of SFV + IAV coinfection at 7 dpi (Supplementary figure 5h). By 10 dpi, effector CD4^+^ T cells in the spleen had a CD25^+^ KLRG1^+^ PD1^+^ triple‐positive and CD25^+^ KLRG1^+^ double‐positive phenotype (Supplementary figure 2h).

### Coinfection changes the relative levels of subpopulations of activated IAV‐ and SFV‐specific CD8 ^+^ T cells in target tissues

**Figure 6:**
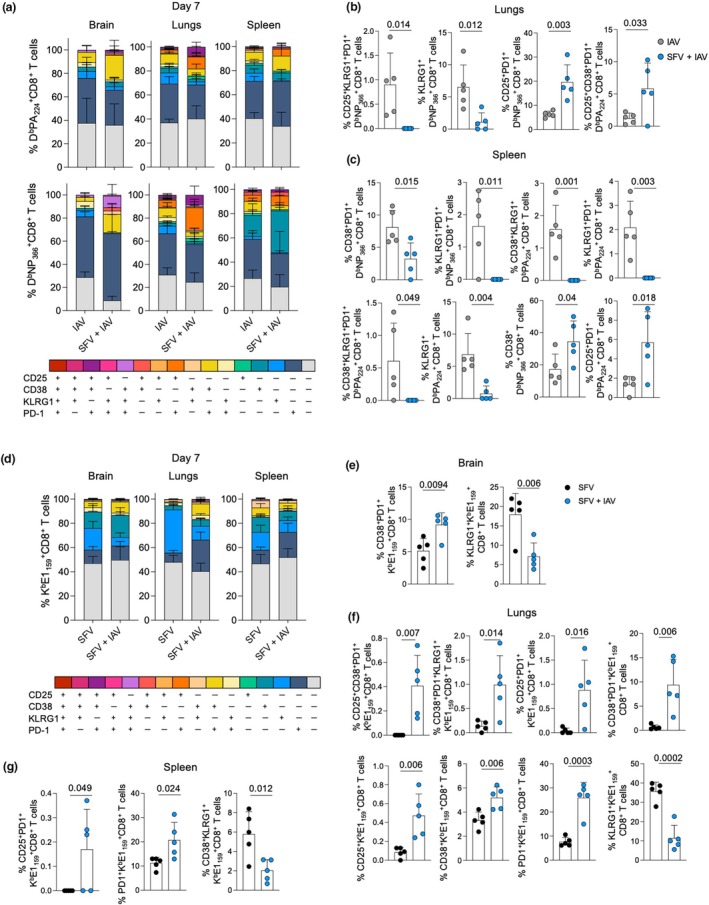
Activation profiles of virus‐specific CD8^+^ T cells across anatomical sites in influenza A virus (IAV)–, Semliki Forest virus (SFV)– and SFV + IAV–infected mice at 7 days postinfection (dpi). Stacked bar graphs depicting frequencies of activation marker combinations on **(a)** D^b^PA_224_^+^CD8^+^ T cells (top row) and D^b^NP_366_^+^CD8^+^ T cells (bottom row) on 7 dpi in IAV‐ and SFV + IAV–infected mice; (*n* = 5, error bar represents standard deviation). Comparison of significantly different activation marker combinations in the **(b)** lungs and **(c)** spleen on D^b^PA_224_^+^CD8^+^ T cells and D^b^NP_366_^+^CD8^+^ T cells on 7 dpi (*n* = 5, error bar represents standard deviation). **(d)** Stacked bar graphs depicting frequencies of activation marker combinations on K^b^E1_159_^+^CD8^+^ T cells on 7 dpi in SFV‐ and SFV + IAV–infected mice; (*n* = 5, error bar represents standard deviation). Comparison of significantly different activation marker combinations in the **(e)** brain, **(f)** lungs and **(g)** spleen on K^b^E1_159_^+^CD8^+^ T cells at 7 dpi (*n* = 5, error bar represents standard deviation). Each symbol denotes an individual mouse. Significance was determined by the Student's unpaired *t*‐test.

As the next step, we focused on activation profiles of virus‐specific CD8^+^ T cells, and examined key T‐cell activation markers (CD25, CD38, KLRG1, PD‐1) in the brain, lungs and spleen of SFV‐, IAV‐ and SFV + IAV–infected mice. Analysis of IAV tetramer^+^CD8^+^ T cells from the lungs revealed that higher proportions of D^b^NP_366_^+^CD8^+^ T cells had CD25^+^KLRG1^+^ and KLRG1^+^ phenotypes in IAV infection, whereas the SFV + IAV group had more CD25^+^PD‐1^+^ D^b^NP_366_^+^CD8^+^ T cells and CD25^+^CD38^+^PD‐1^+^ D^b^PA_224_^+^CD8^+^ T cells in the lungs at 7 dpi (Figure 6a, b). At 10 dpi, higher frequencies of both D^b^NP_366_^+^CD8^+^ T and D^b^PA_224_^+^CD8^+^ T cells were detected in the lungs of coinfected animals displaying more activated phenotypes of CD25^+^CD38^+^PD‐1^+^, CD25^+^CD38^+^, CD25^+^PD‐1^+^ and CD25^+^ (Supplementary figure 3b). In the spleen at 7 dpi, we observed higher proportions of D^b^NP_366_^+^CD8^+^ T cells expressing CD38^+^PD‐1^+^ and KLRG1^+^PD‐1^+^; and higher proportions of D^b^PA_224_^+^CD8^+^ T cells expressing CD38^+^KLRG1^+^, KLRG1^+^PD1^+^, CD38^+^KLRG1^+^PD‐1^+^ and KLRG1^+^ in IAV infection compared with coinfection. However, in the SFV + IAV group, we found higher frequencies of D^b^NP_366_^+^ CD8^+^ T cells with CD38^+^ single‐positive phenotype, and D^b^PA_224_^+^CD8^+^ T cells with CD25^+^PD‐1^+^ activation phenotype (Figure 6c). Conversely, at 10 dpi, most tetramer^+^CD8^+^ T cells in the spleen of SFV + IAV animals had higher activation status compared with IAV‐only infection, expressing activation markers CD25^+^ CD38^+^PD‐1^+^, CD25^+^CD38^+^, CD25^+^PD‐1^+^ and CD38^+^. By contrast, in IAV‐only infection, a higher proportion of tetramer^+^CD8^+^ T cells had CD38^+^PD‐1^+^, CD25^+^CD38^+^PD‐1^+^ and KLRG1^+^ phenotypes (Supplementary figure 3c). Finally, no differences in the activation profiles of D^b^NP_366_^+^CD8^+^ and D^b^PA_224_^+^CD8^+^ T cells in the brains of IAV and SFV + IAV animals were detected (Figure 6a; Supplementary figure 3a).

In‐depth analysis revealed that the expression of CD38^+^PD‐1^+^ was higher, while KLRG1^+^ expression was lower on K^b^E1_159_^+^CD8^+^ T cells in SFV + IAV–infected brain in comparison to SFV‐only infection (Figure 6d, e). Furthermore, K^b^E1_159_^+^CD8^+^ T cells in the lungs of the SFV + IAV group displayed a more activated profile with higher frequencies of CD25^+^CD38^+^PD‐1^+^, CD38^+^PD‐1^+^KLRG1^+^, CD25^+^PD‐1^+^, CD38^+^PD‐1^+^, CD25^+^, CD38^+^ and PD‐1^+^ phenotypes compared with SFV only. However, SFV‐specific CD8^+^ T cells in the lungs of SFV‐only infected mice had greater expressions of KLRG1^+^ (Figure 6f; Supplementary figure 3e). Similarly in the spleen, we found higher frequencies of CD25^+^PD‐1^+^ and PD‐1^+^ SFV‐specific CD8^+^ T cells in coinfection, but the proportion of CD38^+^KLRG1^+^ was higher in SFV‐only infection at 7 dpi (Figure 6g). No differences in the activation status of K^b^E1_159_^+^CD8^+^ T cells between infection types in the brain and spleen at 10 dpi were found (Supplementary figure 3d).

### Coinfection does not impact IAV‐specific CD8 ^+^ T‐cell memory formation

**Figure 7:**
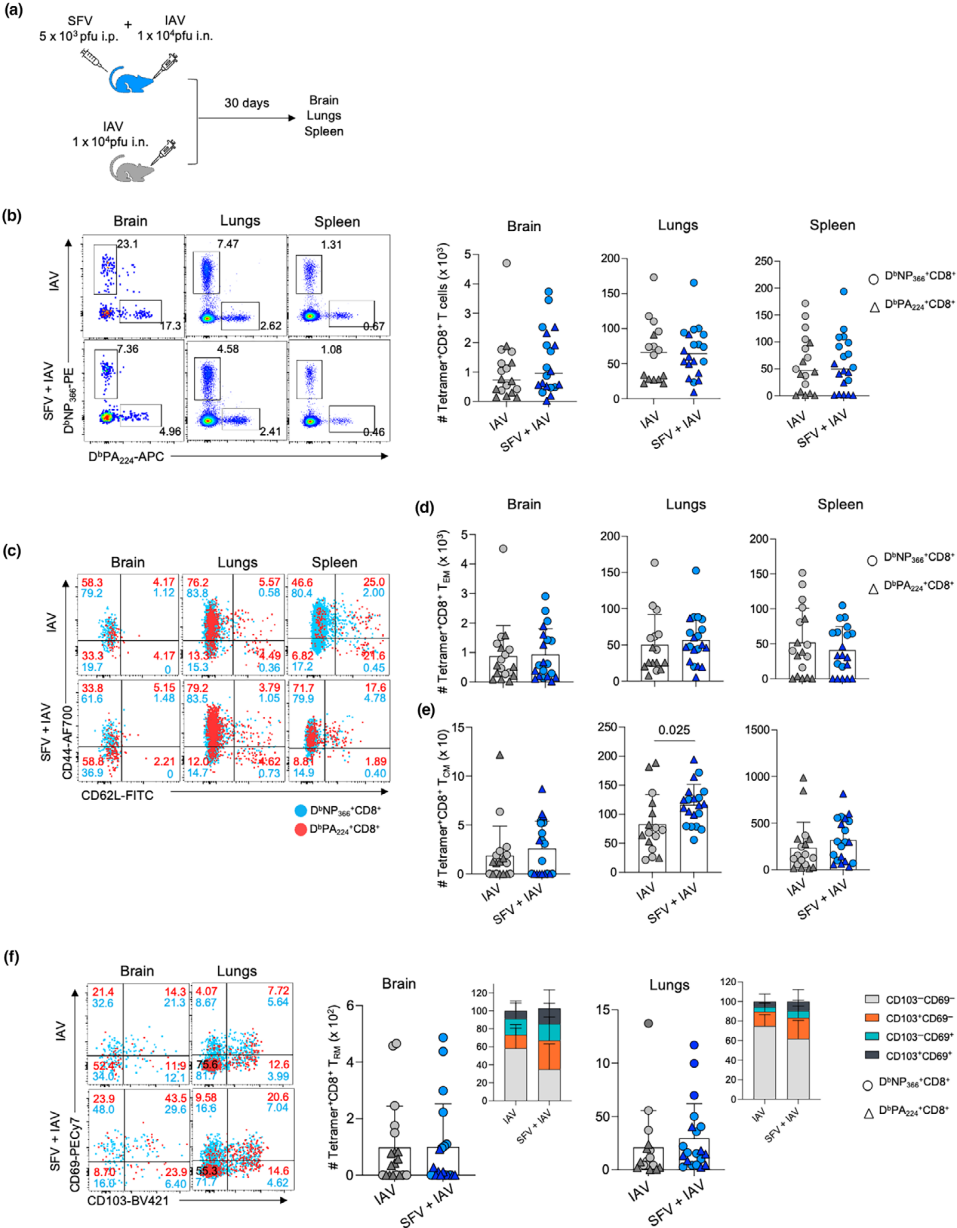
Similar numbers of influenza A virus (IAV)–specific memory CD8^+^ T cells across different anatomical sites in IAV and Semliki Forest virus (SFV) + IAV. **(a)** Mice were infected at the same time with 10^4^ plaque‐forming units (pfu) A/HKx31 [intranasally (i.n.)] and 5 × 10^3^ pfu A7(74) SFV [intraperitoneally (i.p.)]. The IAV‐only group was included as control. Brain, lungs and spleens were harvested at 30 days postinfection (dpi). **(b)** Absolute numbers of IAV‐specific CD8^+^ T cells directed at the D^b^PA_224_ and D^b^NP_366_ epitopes are shown across different anatomical sites in IAV‐ and SFV + IAV–infected mice. Representative fluorescence‐activated cell sorting (FACS) plots are shown for each group and tissue at different time points (*n* = 10, error bars represent median). **(c)** Representative FACS plots are shown for each group and tissue. Absolute numbers of IAV‐specific **(d)** effector memory T cells (T_EM_) and **(e)** central memory T cells (T_CM_) CD8^+^ T cells are shown for D^b^PA_224_^+^CD8^+^ and D^b^NP_366_^+^CD8^+^ T‐cell specificities in the brain, lungs and spleen of IAV‐ and SFV + IAV–infected mice at 30 dpi; (*n* = 10, error bar represents standard deviation). **(f)** Absolute numbers and frequencies of IAV‐specific tissue‐resident memory T cells (T_RM_) CD8^+^ T cells are shown for D^b^PA_224_^+^CD8^+^ and D^b^NP_366_^+^CD8^+^ T‐cell specificities in the brain and lungs of IAV‐ and SFV + IAV–infected mice at 30 dpi. Representative FACS plots are shown for each group and tissue (*n* = 10, two independent experiments, error bar represents standard deviation). Each symbol denotes an individual mouse. Significance was determined by the Student's unpaired *t*‐test.

We demonstrated that simultaneous coinfection of SFV and IAV elicits impaired protective response compared with IAV‐only infection across all tissues in an acute setting. We subsequently investigated the long‐term impact of simultaneous coinfection and the ability to form long‐term tissue‐resident memory T cells (T_RM_) CD8^+^ T‐cell and other CD8^+^ T‐cell memory populations, including central memory T cells (T_CM_) and effector memory T cells (T_EM_) memory pools. Mice were infected with 5 × 10^3^ pfu SFV (A7(74)) intraperitoneally and intranasal with 10^4^ pfu IAV (x31) at the same time. The IAV‐only group was included as a control. Brain, lungs and spleen were collected at 30 dpi (Figure 7a).

At 30 dpi, tetramer^+^CD8^+^ T cells of D^b^NP_366_ and D^b^PA_224_ specificities were detected across all tissues regardless of infection type in similar numbers (Figure 7b). This finding contrasted with the acute setting where the numbers of IAV‐specific CD8^+^ T cells were greater than that of coinfection across all tissues (Figure 4a). More specifically, we observed that SFV + IAV coinfection significantly increases the numbers of T_CM_ in the lungs compared with IAV‐only infection at 30 dpi, albeit no differences were observed in the brain and spleen (Figure 7c, e). In addition, we found that the numbers of IAV‐specific T_EM_ across IAV and SFV + IAV infections in the brain, lungs and spleen were comparable (Figure 7c, d). Furthermore, we detected sizeable populations of IAV‐specific CD8^+^ T‐cell memory at 30 dpi in both IAV‐only and SFV + IAV coinfection across brain and lungs. No differences in the numbers and proportions of IAV‐specific T_RM_ across infection types were found (Figure 7f).

### SFV + IAV and SFV mice exhibit comparable ability to establish resident SFV‐specific CD8 ^+^ T‐cell memory pools

**Figure 8:**
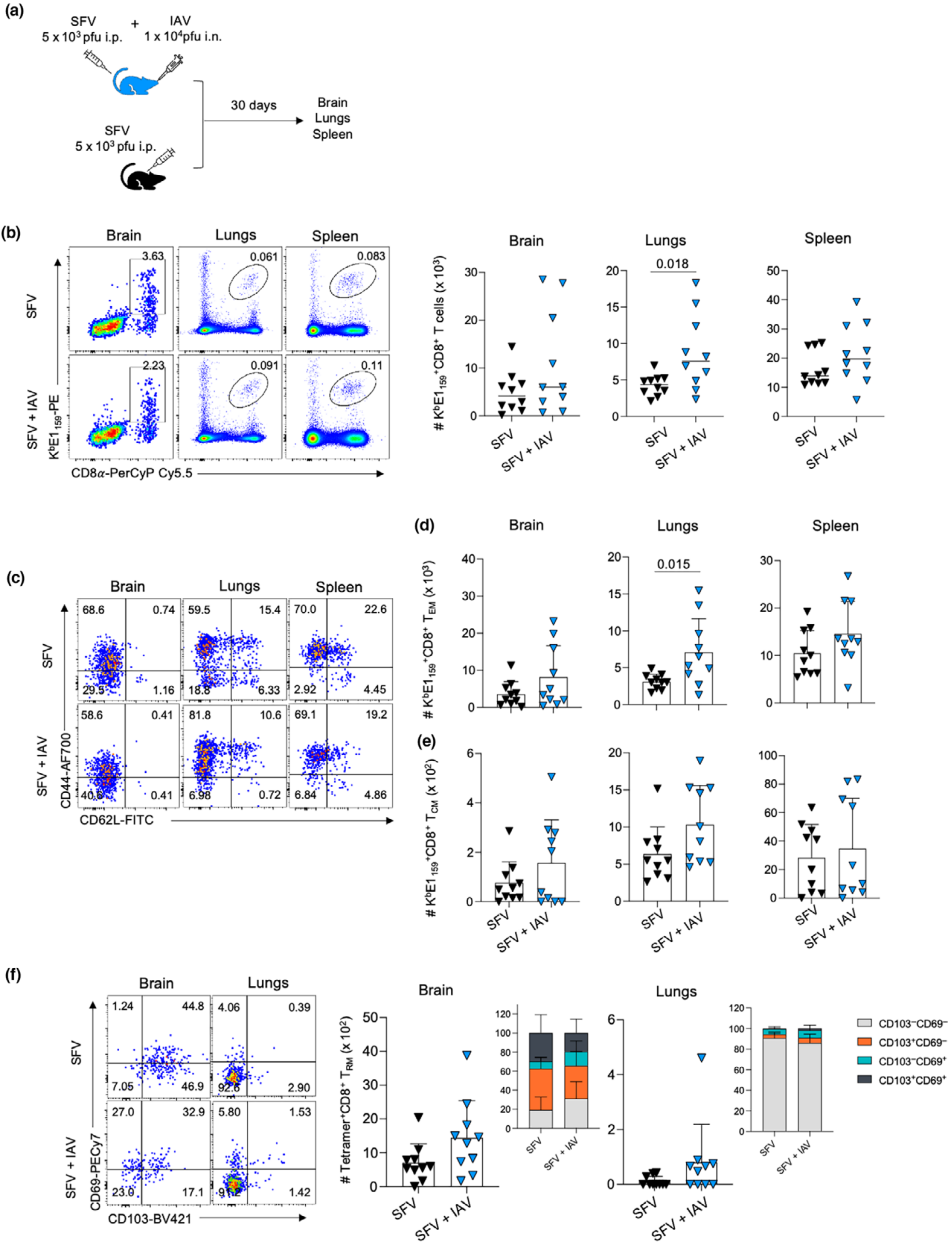
Similar numbers of Semliki Forest virus (SFV)–specific memory CD8^+^ T cells across different anatomical sites in SFV and SFV + influenza A virus (IAV). **(a)** Mice were infected at the same time with 10^4^ plaque‐forming units (pfu) A/HKx31 [intranasally (i.n.)] and 5 × 10^3^ pfu A7(74) SFV [intraperitoneally (i.p.)]. The SFV‐only group was included as control. Brain, lungs and spleens were harvested at 30 days postinfection (dpi). **(b)** Absolute numbers of SFV‐specific CD8^+^ T cells directed at the K^b^E1_159_ epitope are shown across different anatomical sites in SFV‐ and SFV + IAV–infected mice. Representative fluorescence‐activated cell sorting (FACS) plots are shown for each group and tissue at different time points (*n* = 10, error bars represent median). **(c)** Representative FACS plots are shown for each group and tissue. Absolute numbers of SFV‐specific **(d)** effector memory T cells (T_EM_) and **(e)** central memory T cells (T_CM_) CD8^+^ T cells are shown for K^b^E1_159_^+^CD8^+^ T‐cell specificities in the brain, lungs and spleen of SFV‐ and IAV + SFV–infected mice at 30 and 90 dpi (*n* = 10, error bar represents standard deviation). **(f)** Absolute numbers and frequencies of SFV‐specific tissue‐resident memory T cells (T_RM_) CD8^+^ T cells are shown for K^b^E1_159_^+^CD8^+^ T‐cell specificities in the brain and lungs of SFV‐ and SFV + IAV–infected mice at 30 dpi. Representative are plots shown for each group and tissue (*n* = 10, two independent experiments, error bar represents standard deviation). Each symbol denotes an individual mouse. Significance was determined by the Student's unpaired *t*‐test.

We demonstrated that simultaneous coinfection with SFV and IAV elicits similar immune responses compared with SFV‐only infection in an acute setting. We further investigated the long‐term impact of simultaneous coinfection on the generation of long‐term resident CD8^+^ T_RM_ and other CD8^+^ T‐cell memory populations, including CD8^+^ T_CM_ and CD8^+^ T_EM_ memory pools. Mice were infected with 5 × 10^3^ pfu SFV [A7(74)] intraperitoneally and intranasally with 10^4^ pfu IAV (x31) at the same time. The SFV‐only group was included as a control. Brain, lungs and spleen were collected at 30 dpi (Figure 8a). At 30 dpi, tetramer^+^CD8^+^ T cells of K^b^E1_159_ specificity were detected across all tissues regardless of infection type. In accordance with our findings in the acute setting, we observed higher numbers of SFV‐specific CD8^+^ T cells in the lungs of coinfected animals compared with the single‐infection control (Figure 8b). More specifically, we observed significantly higher numbers of SFV‐specific T_EM_ in the lungs of SFV + IAV–infected mice compared with single infection control, but no differences in the numbers of T_CM_ across infection and tissue type (Figure 8c, e). Furthermore, we detected sizeable populations of SFV‐specific CD8^+^ T‐cell memory at 30 dpi in both SFV‐only and SFV + IAV groups across the samples of brain and lungs. However, no differences were detected in the numbers and proportions of SFV‐specific T_RM_ across infection type (Figure 8f).

Overall, our findings demonstrated that simultaneous coinfection of SFV + IAV does not lead to exacerbated or attenuated SFV disease compared with the single infection control group. However, SFV + IAV infection impairs IAV immunity, as demonstrated by increased IAV viral load in the lungs and reduced number of IAV‐specific CD8^+^ T cells in simultaneous coinfection. We postulate that IAV infection does not interfere with SFV immunity in simultaneous coinfection, demonstrated by the minimal changes in SFV immune responses in the brain. Nevertheless, SFV can affect immunity to IAV in simultaneous coinfection as shown by higher viral titers and changes to the cytokine milieu. Presumably, this is a result of SFV transient presence in the lungs on 3 dpi, which could have triggered immune responses inadvertently leading to detrimental changes in anti‐IAV immunity. This is reminiscent to our findings from sequential coinfection with both viruses, although the effects are not as profound. Thus, our current study, together with our previously published observations,^9^ highlights different outcomes of coinfection, implying that the order of coinfection determines disease severity.

## DISCUSSION

Respiratory infections and arboviral diseases are both major global health burdens.^20^ Coinfection in humans of respiratory viruses such as IAV and SARS‐CoV‐2 or of arboviruses such as dengue and Zika is well studied,^21^, ^22^, ^23^ but the consequences of coinfection between unrelated acute viruses, such as a respiratory virus and a neurotropic arbovirus, remain unclear. In previous studies, we have demonstrated that the order of these two infections is critical, producing opposite effects if reversed.^9^ In this study, we show that infecting mice with SFV and IAV at the same time through different routes of infection does not exacerbate disease severity of either virus (Figure 1i, k); however, IAV‐specific T‐cell responses were reduced by coinfection.

The weight loss kinetics following SFV single infection and simultaneous coinfection were markedly different; however, there was no difference between the IAV and SFV + IAV groups. Therefore, the increased body weight loss observed in coinfected mice can be attributed to IAV. SFV infection leads to a marginal weight loss while IAV causes major weight loss and the SFV + IAV infection appears to be intermediate. Thus, although IAV is the major driver of weight loss, this has not been exacerbated by the SFV infection, which is intriguing because these coinfected mice have higher lung IAV infectivity titers and higher levels of inflammatory cytokines and chemokines, which should have led to a greater weight loss, but this is not the case. By contrast, the induction of IFNβ production by a non‐neurotropic strain of influenza (X31) in the brain was surprising. Our previous publication^9^ also showed the presence of matrix (M) antigen in the brain of IAV‐only infected mice, suggesting induction of antiviral responses against IAV antigen in the brain, despite the fact that the infectious virus could not be detected by infectivity assay. However, the level of expression of IFNβ at 3 and 10 dpi was relatively modest, with the mean concentration being 39 and 89 pg mL^−1^ at 3 and 10 dpi, respectively.

One might expect that a more rapid and systemic activation of innate immune responses would hinder IAV replication in the lungs, leading to reduced viral replication and faster clearance upon coinfection with SFV. However, our data presented in Figure 1d demonstrated that IAV viral titer were comparable in both IAV and SFV + IAV groups on 3 dpi. This implies that even if SFV infection induced a more rapid activation of innate immune responses, it did not affect the initial replication of IAV. However, the key difference was observed on 7 dpi, when the SFV + IAV group exhibited significantly lower lung IAV viral titers compared with the IAV‐only group. In addition, higher levels of cytokines and chemokines suggest dysregulated production of immunomodulators (Figure 2c). At the peak of the adaptive response on day 10, we observed a significantly lower number of IAV‐specific cells in the lungs of the SFV + IAV group (Figure 4b). These findings suggest that SFV exerts its negative effect on anti‐IAV immunity later in the infection.

IAV‐specific CD8^+^ T‐cell responses were affected in simultaneous coinfection as demonstrated by the reduced numbers of IAV‐specific T cells across all tissues in coinfection relative to IAV‐only infection. The higher IAV viral titer observed at 7 dpi in the coinfected mice could, in part, be explained by the lower number of IAV‐specific CD8^+^ T cells in the lungs, leading to delayed viral clearance. This reduced T‐cell response could result from antigenic competition, where immune responses to one determinant are hindered by coexposure to others; the increased variety of antigens in simultaneous coinfection increased MHC competition within APCs and reduced the efficiency of p‐MHC presentation for each virus. Conversely, numbers of SFV‐specific CD8^+^ T cells in the lungs of SFV + IAV–coinfected mice are higher than in the SFV single infection. This could result from the higher levels of lung cytokines and chemokines induced by the IAV infection promoting the expansion of SFV‐specific CD8^+^ T cells. SFV‐specific T‐cell responses in the brain were unaffected by coinfection and SFV infectivity titers in the brain and brain pathology were unchanged by coinfection.

Analysis of different T‐cell activation markers indicated that higher proportions of bulk effector CD8^+^ and CD4^+^ T cells express CD38^+^ PD‐1^+^ and CD38^+^ activation profiles in the brain, lungs and spleen of SFV + IAV–coinfected animals at 7 dpi. Coexpression of CD38 and PD1 on CD8^+^ T cells has been reported previously to be associated with higher viral load and T‐cell hyperactivation at the site of infection in severe influenza disease.^9^, ^13^ This finding suggests that the inflammatory environment in the periphery (i.e. spleen and draining lymph nodes) recruits activated bystander CD8^+^ T cells to the site of infection (lungs) as well as to the brain. Coexpression of CD38 and PD‐1 on CD8^+^ T cells of SFV + IAV mice may also suggest hyperactivation of T cell because of the need to control infections from two invading pathogens.^13^, ^24^, ^25^ By contrast, CD25 expression was the common denominator on IAV tetramer^+^CD8^+^ T cells in the lungs and spleen of coinfected animals, a marker associated with early activation in IAV infection.^26^ However, because CD25 is upregulated at 10 dpi in SFV + IAV, it can be postulated that IAV‐specific CD8^+^ T cells in coinfection had delayed activation, leading to higher viral load at the site of infection. IAV‐specific CD8^+^ T cells were observed to traffic to the brain in both IAV and SFV + IAV groups. No differences in activation profiles of these cells were observed.

We demonstrated that following SFV + IAV coinfection, coinfected mice could respond at the same capacity as IAV‐only and SFV‐only infected animals at 30 dpi across tissues, implicating that simultaneous coinfection does not affect the formation of virus‐specific memory T cells (T_EM_, T_CM_ and T_RM_). Interestingly, lung numbers of SFV‐specific CD8^+^ T cells and SFV‐specific T_EM_ were higher in the SFV + IAV group compared with the SFV‐only group. This was also observed in the acute setting where higher numbers of SFV‐specific CD8^+^ T cells in the lungs of the SFV + IAV group were detected. These SFV‐specific CD8^+^ T cells then underwent contraction in a comparable manner to effector memory T cells. While we observed differences in CD8^+^ T‐cell responses during the acute phase in simultaneous coinfection, no differences in CD8^+^ T‐cell memory formation for IAV‐specific responses were detected.

Taken together, the order and timing of coinfection with two unrelated acute viruses can alter the disease phenotype and underpinning immunity. When mice were infected with SFV before IAV, it resulted in severe respiratory disease.^9^ Conversely, mice infected with IAV followed by SFV had mild neurotropic disease in comparison to the single infection control. In this study, we demonstrated that simultaneous coinfection with SFV + IAV perturbs influenza‐specific responses but does not lead to exacerbated lung disease. Our data indicate that IAV does not interfere with SFV immunity in simultaneous coinfection, as minimal changes in SFV immune responses in the brain were detected. Regardless, SFV appears to hamper IAV antigen presentation in the coinfection setting.

## METHODS

### Ethics statement

C57BL/6J mice were bred at the Peter Doherty Institute Bioresources Facilities. Animal experiments followed the National Health and Medical Research Council (NHMRC) Code of Practice for the Care and Use of Animals for Scientific Purposes guidelines and were approved by the University of Melbourne Animal Ethics Committee (AEC 1714184 and 21319). All mice were monitored daily for clinical sign including determination of their body weight.

### Viruses, virus infection and measurement

The influenza virus strain A/X31(H3N2) used in this study was grown in the allantoic cavity of 10‐day‐old embryonated chicken eggs in accordance with World Health Organization guidelines and ethics (WHO Flu AEC 27270). The titer of infectious virus was determined by plaque assay on monolayers of Madin–Darby canine kidney cells as described previously.^13^ The avirulent A7(74) strain of SFV was generated from the plasmid pCMV‐SFVA774‐2SG provided by Professor Andres Merits (University of Tartu, Institute of Technology, Tartu, Estonia). This plasmid expresses the viral genomic RNA under the control of a cytomegalovirus promoter. SFV was grown in baby hamster kidney fibroblasts.^9^, ^27^ A Vero cell plaque assay was used to titrate infectious SFV, as described previously.^27^

C57BL/6J mice were used in our study as these mice are well characterized for both influenza and SFV infection models. Importantly, immunodominant influenza‐specific and SFV‐specific CD8^+^ T‐cell epitopes have been identified and well characterized in C57BL/6J mice, namely D^b^NP_366_ and D^b^PA224 (influenza) and K^b^E1_159_ (SFV). Mice (6–8 weeks old) were inoculated intraperitoneally with 5 × 10^3^ pfu of the avirulent A7(74) strain of SFV in 0.1 mL phosphate‐buffered saline with albumin. For influenza virus infections, mice were lightly anesthetized with isoflurane (Veterinary Companies of Australia, Kings Park, NSW, Australia) using an anesthetic machine (Advanced Anesthesia Specialists, Sydney, NSW, Australia) and infected by intranasal instillation of 10^4^ pfu of x31 [A/x31 (H3N2)] influenza virus in 30 μL phosphate‐buffered saline.

### Tissue sampling and single‐cell preparation

Brain, lungs and spleen were collected from mice at various time points after infection. To remove blood from the tissue vasculature, animals were perfused with 10 mL phosphate‐buffered saline through the left cardiac ventricle following terminal anesthesia. Brains were removed and processed for virus infectivity assay, measurement of chemokine/cytokine levels (in each case a half brain bisected sagittally along the midline), preparation of RNA for gene expression studies (half brain) or analysis of cell infiltrates (entire brain). Single‐cell suspensions were purified from the brain, lungs and spleen. The brain tissue was digested with 1785 units mL^−1^ collagenase type III (Worthington Biochemical Corporation, Lakewood, NJ, USA) and 6 units mL^−1^ DNase I (Sigma ‐Aldrich, St. Louis, MO, USA). Central nervous system–infiltrating leukocytes were isolated from brain samples by centrifugation on a Percoll (Sigma ‐ Aldrich, St. Louis, MO, USA) gradient (70%, 37% and 30% Percoll). Lungs were either homogenized and centrifuged to obtain clarified supernatants to assay for viral titers/cytokine composition or enzymatically digested in 1 mg mL^−1^ collagenase III (Worthington) and 0.5 mg mL^−1^ DNase I (Sigma‐Aldrich) before passing through cell sieves to obtain single‐cell suspensions for analysis. Where necessary, cell suspensions from tissues were incubated with 0.15 M NH_4_Cl and 17 mM Tris‐HCI at pH 7.2 for 5 min at 37°C to lyse red blood cells.

### Tetramer and phenotypic staining

MHC‐I tetramers targeting the immunodominant epitope of the SFV envelope protein (K^b^‐E1_159‐166_: TQFIFGPL) and influenza nucleoprotein (D^b^NP_366–374_: ASNENMETM; D^b^PA_224–233_: SSLENFRAYV) were produced in‐house and conjugated to streptavidin–APC (catalog number 554067; BD Biosciences, New Jersey, USA), PE (catalog number 554061; BD Biosciences) and BV421 (catalog number 563259; BD Biosciences), respectively, at 1:200 dilution at room temperature for 1 h in the dark. A 1:4 ratio of streptavidin to biotinylated MHC‐I protein was used and the streptavidin was added to the biotinylated MHC‐I protein in 10 increments, with a 10‐minute interval between each addition.

Lymphocytes were stained with combinations of fluorochrome‐conjugated antibodies: BD Biosciences (New Jersey, USA): anti‐CD8α‐PerCyP Cy5.5 (clone 53–67; catalog number 551162), anti‐CD44‐Alexa Flour 700 (clone 1M7; catalog number 560567), anti‐CD4‐APC Cy7 (clone GK1.5; catalog number 552051), anti‐TCRβ‐BV711 (clone H57‐597; catalog number 563135), anti‐CD25‐PECF594 (clone PC61; catalog number 562694), anti‐CD45.1‐FITC (clone A20; catalog number 561871), anti‐CD62L‐FITC (clone MEL‐14; catalog number 561917), anti‐CD45.1‐PE (clone A20; catalog number 553776), anti‐V2αTCR‐FITC (clone B20.1; catalog number 553288), anti‐CD45R‐APCCy7 (clone RA3‐6B2; catalog number 552094), anti‐CD38‐BV711 (clone 90; catalog number 740697), anti‐CD138‐PE (clone 281–2; catalog number 553714), anti‐CD11c‐FITC (clone HL3; catalog number 557400), anti‐Gr1‐FITC (clone RB6‐8C5; catalog number 553126), anti‐CD64‐AF647 (clone X54‐5/7.1; catalog number 558539), anti‐Ly6C‐AlexaFlour700 (clone AL‐21; catalog number 561237), anti‐CD11b‐BV605 (clone M1/70; catalog number 563 015), anti‐CD45.2‐BV711 (clone 104; catalog number 563685), anti‐CD11c‐PE (clone HL3; catalog number 553802), anti‐SigLecF‐PECF594 (clone E50‐2440; catalog number 562757), anti‐Ly6G‐PECy7 (clone 1A8; catalog number 560601). BioLegend (San Diego, CA, USA): anti‐CD62L‐BV570 (clone MEL‐14; catalog number 104433), anti‐CD279‐BV785 (clone 29F.1A12; catalog number 135225), anti‐CD38‐PECy7 (clone 90; catalog number 102718), anti‐CD8α‐BV510 (clone 53–6.7; catalog number 100752), anti‐CD103‐BV421 (clone 2E7; catalog number 121422), anti‐CD69‐PECy7 (clone H1.2F3; catalog number 104512), anti‐CD62L‐PECy7 (clone MEL‐14; catalog number 104418), anti‐GL7‐PerCyPCy5.5 (clone GL7; catalog number 144610), anti‐CD19‐APC (clone 6D5; catalog number 11512), anti‐I‐Ab‐PacBlue (clone AF6‐120.1; catalog number 116422), anti‐IgD‐PECy7 (clone 11‐26c.2a; catalog number 405720), anti‐CD3‐FITC (clone 145‐2C11; catalog number 100306) and anti‐F4/80‐FITC (clone RB6‐8C5; catalog number 553126). Invitrogen eBiosciences (Waltham, Massachusetts, USA) anti‐KLRG1‐FITC (clone 2F1; catalog number 11–5893‐82). Cell viability was determined by staining with either Live/Dead‐Aqua 525 (L34966A; Thermo Fisher, Waltham, MA, USA) or Live/Dead Fixable Near‐IR (L10119, Thermo Fisher, Waltham, MA, USA).

Cells were fixed with 1% paraformaldehyde before analysis by flow cytometry. Antibody staining was performed at 4°C in the dark. Samples were subsequently acquired on a BD LSR Fortessa (BD Biosciences, Franklin Lakes, NJ, USA) flow cytometer and data analyzed by FlowJo 10.10 Software (Tree Star Inc., Ashland, OR, USA). Gating strategies for flow cytometry data are as described in Supplementary figure 4.

### Cytokine analyses

Cytokine and chemokine levels in lungs and brain homogenates were analyzed using the LEGENDPlex Multi‐Analyte Flow Assay Kit (BioLegend, San Diego, CA, USA) Mouse Anti‐Virus Response Panel (13‐plex) according to the manufacturers’ instructions.

### Histopathology

Lungs and brains were removed from phosphate‐buffered saline–perfused animals, fixed in 4% paraformaldehyde in phosphate‐buffered saline, processed for embedding in paraffin wax, cut into thin sections, stained with hematoxylin and eosin and analyzed microscopically. Multiple sections were cut through the lungs at 5‐μm thickness at 100‐μm intervals (5× levels), while the brains were cut through at 5‐μm thickness at 50‐μm intervals (5× levels).

Histopathology and grading were performed by the Phenomics Australia Histopathology and Digital Slide Service at the University of Melbourne. The scope of grading for the brain samples included neuronal cell loss, degeneration and necrosis, neural dysplasia, angiectasis, axonopathy, epidermoid cysts, capillary injury and hemorrhage, hydrocephalus, inflammation, microgliosis, spongiform change of white matter with myelin breakdown, mineralization, pigment and vascular reaction‐endothelial hypertrophy/hyperplasia. Standard histological assessment of lungs covered the following: atelectasis, alveolar proteinosis, alveolar septal thickening, hyaline membranes, intra‐alveolar and interstitial edema, prominent type II pneumocyte proliferation, interstitial congestion, intra‐alveolar hemorrhage, inflammation, presence of pathogens or exogenous material, fibrosis, hyperplasia/regeneration of bronchial/bronchiolar epithelium, degeneration or loss of bronchial/bronchiolar epithelium and alveolar necrosis.

A grading scale ranging from 0 to 3 was used for both lungs and brains to demonstrate degrees of change:

- 0 = no changes/mild changes considered insignificant.
- 1 = minimal lesions affecting 1–25% of the area.
- 2 = multifocal lesions affecting 25–50% of the area.
- 3 = severe tissue changes affecting > 50% of the area.

Values used to plot damage in Figure 1 are provided in Supplementary table 1.

### Statistical analyses

Significance tests were performed using the Student's unpaired *t*‐test or Tukey's multiple comparison test for comparison between two groups or among multiple groups, respectively, within GraphPad Prism 10.4.1 software (La Jolla, CA, USA).

## AUTHOR CONTRIBUTIONS

**Isabelle Jia‐Hui Foo:** Conceptualization; formal analysis; investigation; methodology; writing – original draft; writing – review and editing. **Aira F Cabug:** Investigation. **Brad Gilbertson:** Resources; writing – review and editing. **John K Fazakerley:** Conceptualization; funding acquisition; resources; supervision; writing – review and editing. **Katherine Kedzierska:** Conceptualization; funding acquisition; resources; supervision; writing – review and editing. **Lukasz Kedzierski:** Conceptualization; formal analysis; investigation; project administration; supervision; writing – review and editing.

## CONFLICT OF INTEREST

All authors declare no competing interests.

## Supporting information


Supplementary figure 1.
Supplementary figure 2.
Supplementary figure 3.
Supplementary figure 4.
Supplementary figure 5.
Supplementary table 1.

## Data Availability

The published article includes all datasets generated or analyzed during the study.

## References

[1] Rückert C , Weger‐Lucarelli J , Garcia‐Luna SM , *et al*. Impact of simultaneous exposure to arboviruses on infection and transmission by Aedes aegypti mosquitoes. Nat Commun 2017; 8: 15412. 28524874 10.1038/ncomms15412 PMC5454532

[2] Bicudo N , Bicudo E , Costa JD , Castro JALP , Barra GB . Co‐infection of SARS‐CoV‐2 and dengue virus: a clinical challenge. Braz J Infect Dis 2020; 24: 452–454. 32866435 10.1016/j.bjid.2020.07.008 PMC7448779

[3] Masyeni S , Santoso MS , Widyaningsih PD , *et al*. Serological cross‐reaction and coinfection of dengue and COVID‐19 in Asia: experience from Indonesia. Int J Infect Dis 2021; 102: 152–154. 33115680 10.1016/j.ijid.2020.10.043 PMC7585717

[4] Kedl RM , Rees WA , Hildeman DA , *et al*. T cells compete for access to antigen‐bearing antigen‐presenting cells. J Exp Med 2000; 192: 1105–1114. 11034600 10.1084/jem.192.8.1105 PMC2195874

[5] Kenney LL , Cornberg M , Chen AT , Emonet S , de la Torre JC , Selin LK . Increased immune response variability during simultaneous viral coinfection leads to unpredictability in CD8^+^ T cell immunity and pathogenesis. J Virol 2015; 89: 10786–10801. 26269191 10.1128/JVI.01432-15 PMC4621125

[6] Esper FP , Spahlinger T , Zhou L . Rate and influence of respiratory virus co‐infection on pandemic (H1N1) influenza disease. J Infect 2011; 63: 260–266. 21546090 10.1016/j.jinf.2011.04.004 PMC3153592

[7] Vidor E . The nature and consequences of intra‐ and inter‐vaccine interference. J Comp Pathol 2007; 137: S62–S66. 17560593 10.1016/j.jcpa.2007.04.014

[8] Ruben FL , Smith EA , Foster SO , *et al*. Simultaneous administration of smallpox, measles, yellow fever, and diphtheria—pertussis—tetanus antigens to Nigerian children. Bull World Health Organ 1973; 48: 175–181. 4541683 PMC2481001

[9] Foo IJ‐H , Chua BY , Clemens EB , *et al*. Prior infection with unrelated neurotropic virus exacerbates influenza disease and impairs lung T cell responses. Nat Commun 2024; 15: 2619. 38521764 10.1038/s41467-024-46822-7 PMC10960853

[10] Bradish CJ , Allner K . The early responses of mice to respiratory or intraperitoneal infection by defined virulent and avirulent strains of Semliki Forest virus. J Gen Virol 1972; 15: 205–218. 5040350 10.1099/0022-1317-15-3-205

[11] Rodriguez‐Madoz JR , Prieto J , Smerdou C . Biodistribution and tumor infectivity of Semliki Forest virus vectors in mice: effects of Re‐administration. Mol Ther 2007; 15: 2164–2171. 17667947 10.1038/sj.mt.6300274

[12] Kraaijeveld CA , Harmsen M , Khader Boutahar‐Trouw B . Cellular immunity against Semliki Forest virus in mice. Infect Immun 1979; 23: 213–218. 311341 10.1128/iai.23.2.213-218.1979 PMC414150

[13] Jia X , Chua BY , Loh L , *et al*. High expression of CD38 and MHC class II on CD8^+^ T cells during severe influenza disease reflects bystander activation and trogocytosis. Clin Transl Immunol 2021; 10: e1336. 10.1002/cti2.1336 PMC8426257 34522380

[14] Kedzierski L , Tan AEQ , Foo IJH , Nicholson SE , Fazakerley JK . Suppressor of cytokine Signalling 5 (SOCS5) modulates inflammatory responses during alphavirus infection. Viruses 2022; 14: 2476. 36366574 10.3390/v14112476 PMC9692489

[15] Kedzierski L , Er Qi Tan A , Jia Hui Foo I , *et al*. In Semliki Forest virus encephalitis, suppressor of cytokine signaling 4 (SOCS4) is an essential modulator of immune responses that mediates the balance between immunopathology and virus clearance. Immunol Cell Biol 2023; 101: 333–344. 36702633 10.1111/imcb.12625

[16] Fazakerley JK , Webb HE . Semliki Forest virus induced, immune mediated demyelination: the effect of irradiation. Br J Exp Pathol 1987; 68: 101–113. 3028463 PMC2012988

[17] Fragkoudis R , Breakwell L , McKimmie C , *et al*. The type I interferon system protects mice from Semliki Forest virus by preventing widespread virus dissemination in extraneural tissues, but does not mediate the restricted replication of avirulent virus in central nervous system neurons. J Gen Virol 2007; 88: 3373–3384. 18024907 10.1099/vir.0.83191-0

[18] Wang Z , Zhang A , Wan Y , *et al*. Early hypercytokinemia is associated with interferon‐induced transmembrane protein‐3 dysfunction and predictive of fatal H7N9 infection. Proc Natl Acad Sci U S A 2014; 111: 769–774. 24367104 10.1073/pnas.1321748111 PMC3896201

[19] Kedzierska K , Venturi V , Field K , Davenport MP , Turner SJ , Doherty PC . Early establishment of diverse T cell receptor profiles for influenza‐specific CD8^+^CD62L^h^ ^i^ memory T cells. Proc Natl Acad Sci U S A 2006; 103: 9184–9189. 16754852 10.1073/pnas.0603289103 PMC1482587

[20] Girard M , Nelson CB , Picot V , Gubler DJ . Arboviruses: a global public health threat. Vaccine 2020; 38: 3989–3994. 32336601 10.1016/j.vaccine.2020.04.011 PMC7180381

[21] Estofolete CF , Terzian ACB , Colombo TE , *et al*. Co‐infection between zika and different dengue serotypes during DENV outbreak in Brazil. J Infect Public Health 2019; 12: 178–181. 30301701 10.1016/j.jiph.2018.09.007

[22] Alosaimi B , Naeem A , Hamed ME , *et al*. Influenza co‐infection associated with severity and mortality in COVID‐19 patients. Virol J 2021; 18: 127. 34127006 10.1186/s12985-021-01594-0 PMC8200793

[23] Ozaras R , Cirpin R , Duran A , *et al*. Influenza and COVID‐19 coinfection: report of six cases and review of the literature. J Med Virol 2020; 92: 2657–2665. 32497283 10.1002/jmv.26125

[24] Krnic EK , Gagro A , Drazenovic V , *et al*. Enumeration of haemagglutinin‐specific CD8^+^ T cells after influenza vaccination using MHC class I peptide tetramers. Scand J Immunol 2008; 67: 86–94. 18052968 10.1111/j.1365-3083.2007.02042.x

[25] Doisne J‐M , Urrutia A , Lacabaratz‐Porret C , *et al*. CD8^+^ T cells specific for EBV, cytomegalovirus, and influenza virus are activated during primary HIV infection. J Immunol 2004; 173: 2410–2418. 15294954 10.4049/jimmunol.173.4.2410

[26] Keating R , Morris MY , Yue W , *et al*. Potential killers exposed: tracking endogenous influenza‐specific CD8^+^ T cells. Immunol Cell Biol 2018; 96: 1104–1119. 29972699 10.1111/imcb.12189 PMC6282960

[27] Fragkoudis R , Dixon‐Ballany CM , Zagrajek AK , Kedzierski L , Fazakerley JK . Following acute encephalitis, Semliki Forest virus is undetectable in the brain by infectivity assays but functional virus RNA capable of generating infectious virus persists for life. Viruses 2018; 10: 273. 29783708 10.3390/v10050273 PMC5977266

