# Supplementary material for: Simultaneous coinfection with influenza virus and an arbovirus impedes influenza‐specific but not Semliki Forest virus–specific responses
Source: Immunol Cell Biol. 2025 Feb 19;103(4):383–400. doi: 10.1111/imcb.70003 (PMC11964787; doi:10.1111/imcb.70003)
Supplement: Supplementary file 1 — Supplementary figure 1. Supplementary figure 2. Supplementary figure 3. Supplementary figure 4. Supplementary figure 5. Supplementary table 1. [file IMCB-103-383-s001.pdf]

Supporting information

Simultaneous co-infection with influenza virus and an arbovirus impedes influenza-specific but not Semliki Forest virus-specific responses

Isabelle Jia-Hui Foo[1], Aira F Cabug[1], Brad Gilbertson[1], John K Fazakerley[1,2*], Katherine Kedzierska[1#*] and Lukasz Kedzierski[1#*]

[1]Department of Microbiology and Immunology, The University of Melbourne, at the Peter Doherty Institute for Infection and Immunity, Melbourne, VIC 3000, Australia
[2]Department of Veterinary Biosciences, Faculty of Science, University of Melbourne, Melbourne, VIC 3000, Australia

**These authors contributed equally to this study*
#Correspondance: lukaszk@unimelb.edu.au, kkedz@unimelb.edu.au

Running title: IAV and SFV co-infection hampers IAV response

Key words:
Immunity to viral co-infections, influenza virus, arbovirus, encephalitis, central nervous system, CD8$^+$ T cells, innate immunity, inflammation, Semliki Forest virus

Supplementary figure 1. Analysis of innate immune cells and humoral cellular infiltrates in IAV, SFV, and SFV+IAV infected mice at 3, 7, and 10 dpi. Absolute number of **(a)** neutrophils ($CD11b^{+}Ly6G^{+}$), **(b)** macrophages ($CD11b^{+}CD64^{+}F4/80^{+}$), **(c)** pan dendritic cells ($CD11c^{+}$), **(d)** inflammatory monocytes, **(e)** alveolar macrophages (lungs only; $CD64^{+}CD11c^{+}$) , **(f)** B cells ($B220^{+}CD19^{+}$) and **(g)** antibody secreting cells (ASC, IgD^{-} $B220^{lo}CD138^{+}$) in the brain, lungs, and spleen of IAV, SFV, and SFV+IAV infected mice across different timepoints (n=5-10, two independent experiments, error bars represent SD). Significance was determined by Tukey's multiple comparison test.

Supplementary figure 2. Activation profiles of effector $CD4^{+}$ T cells and $CD8^{+}$ T cells across anatomical sites in IAV, SFV, and SFV+IAV infected mice at 10 dpi. Stacked bar graphs depicting frequencies of activation marker combinations on **(a)** $CD8^{+}$ T cells and **(e)** $CD4^{+}$ T cells on 10 dpi in IAV, SFV, and SFV+IAV infected mice; (n=8-10, two independent experiments, error bar represents SD). Comparison of significantly different activation marker combinations in the brain, lungs, and spleen on $CD8^{+}$ T cells **(b, c, d)** and $CD4^{+}$ T cells **(f, g, h)** on 10 dpi (n=8-10, two independent experiments, error bar represents SD). Each symbol denotes an individual mouse. Significance was determined by Tukey's multiple comparisons test.

Supplementary figure 3. Activation profiles of virus-specific $CD8^{+}$ T cells across anatomical sites in IAV, SFV, and SFV+IAV infected mice at 10 dpi. Stacked bar graphs depicting frequencies of activation marker combinations on **(a)** $D^{b}PA_{224}{}^{+}CD8^{+}$ T cells (top row) and $D^{b}NP_{366}{}^{+}CD8^{+}$ T cells (bottom row) on 10 dpi in IAV and SFV+IAV infected mice; (n=8-10, two independent experiments, error bar represents SD). Comparison of significantly different activation marker combinations in the **(b)** lungs and **(c)** spleen on $D^{b}PA_{224}{}^{+}CD8^{+}$ T cells and $D^{b}NP_{366}{}^{+}CD8^{+}$ T cells on 10 dpi (n=8-10, two independent experiments, error bar represents SD). **(d)** Stacked bar graphs depicting frequencies of activation marker combinations on $K^{b}E1_{159}{}^{+}CD8^{+}$ T cells on 10 dpi in SFV and SFV+IAV infected mice; (n=8-10, two independent experiments, error bar represents SD). Comparison of significantly different activation marker combinations in the **(e)** lungs on $K^{b}E1_{159}{}^{+}CD8^{+}$ T cells at 10 dpi (n=8-10, two independent experiments, error bar represents

SD). Each symbol denotes an individual mouse. Significance was determined by the Student's unpaired *t*-test.

Supplementary figure 4. Gating strategy for flow cytometric analysis of lymphocytes in the brain, lungs, and spleen. Antibodies against surface markers (see Methods section) were used in combination in 3 panels to gate on **(a)** myeloid cells, **(b)** B cells, **(c)** T cells, and **(d)** virus-specific memory T cells. Values indicate the percentage of gated or positive cells.

Supplementary figure 5. (a) Representative FACS plots show proportion of IAV-specific $CD8^+$ T cells of D^bNP_{366} and D^bPA_{224} specificities across different tissues in SFV infected mice at 7 and 10 dpi. **(b)** Representative FACS plots show proportion of SFV-specific $CD8^+$ T cells of K^bE1_{159} specificity across different tissues in IAV infected mice at 7 and 10 dpi.

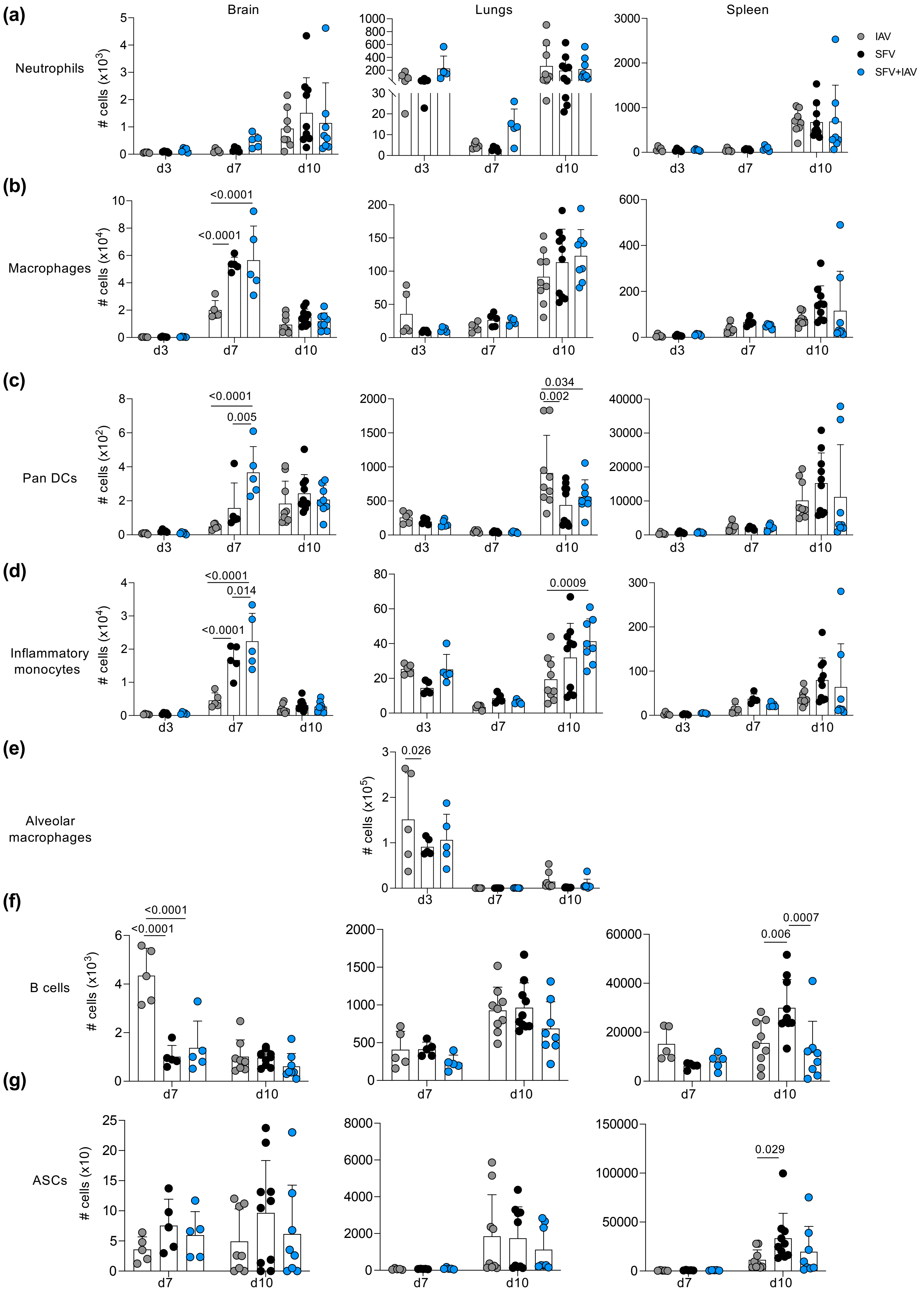

Supplementary figure 1 Foo et al

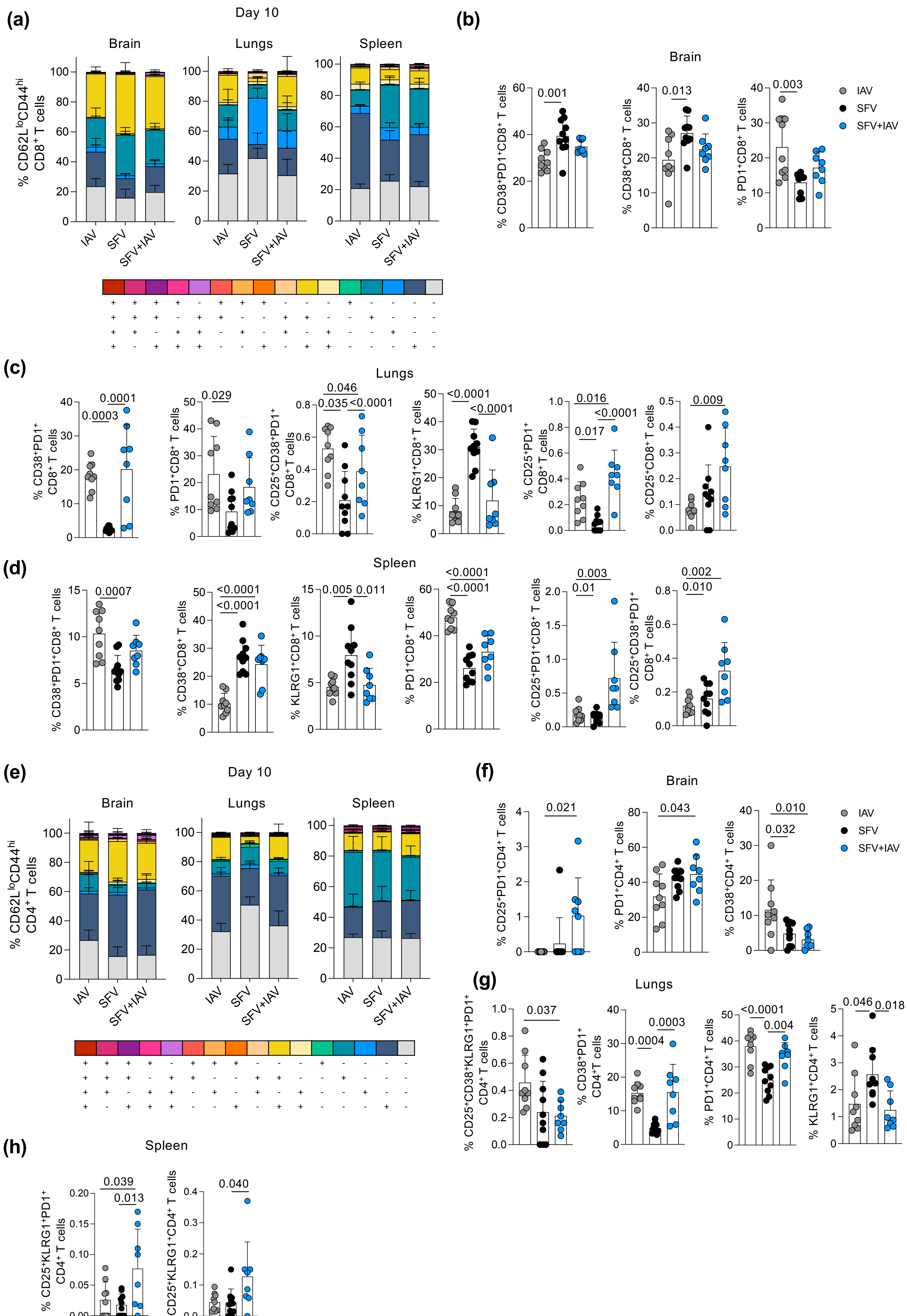

Supplementary figure 2 Foo et al.

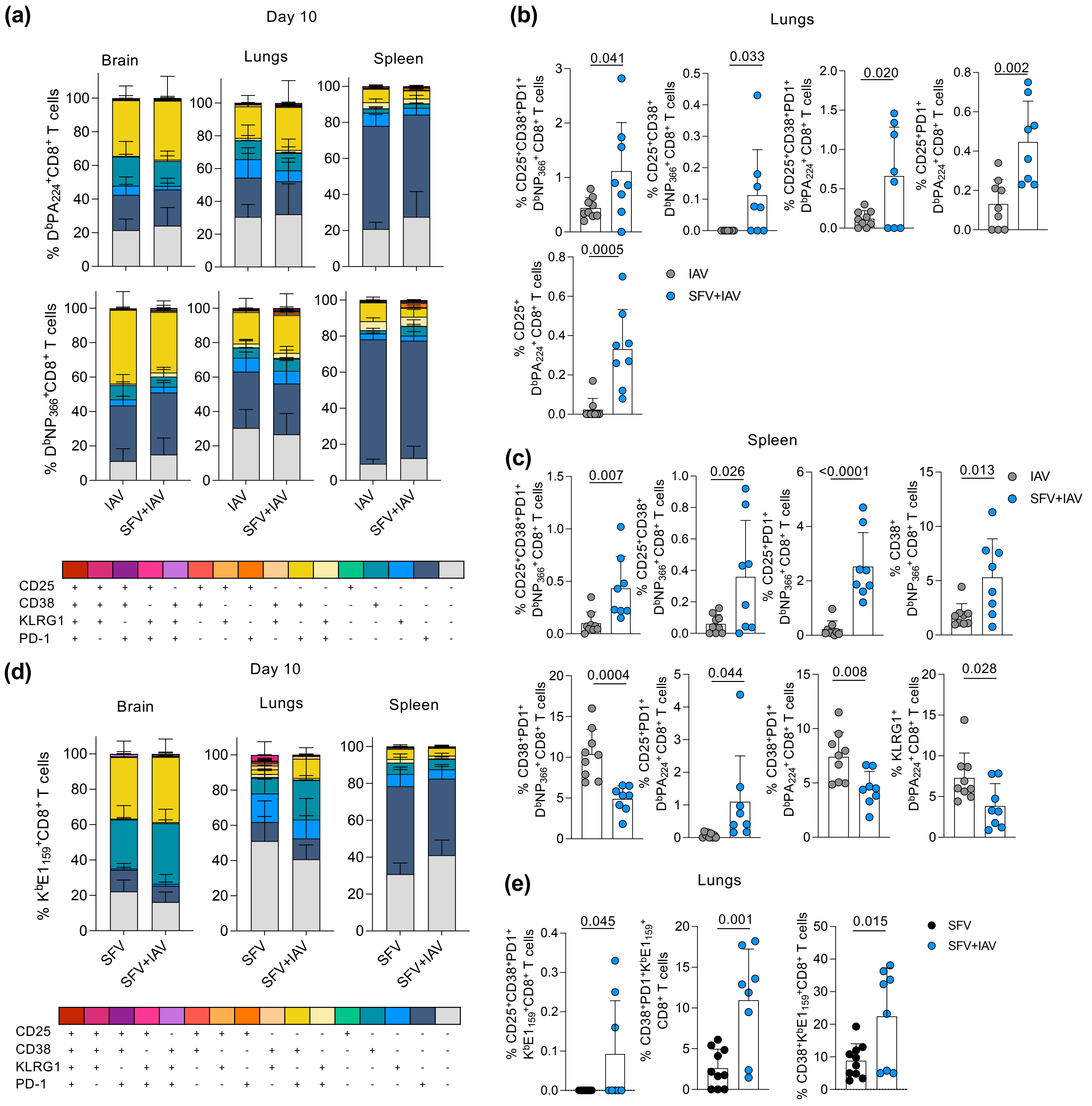

Supplementary figure 3 Foo et al.

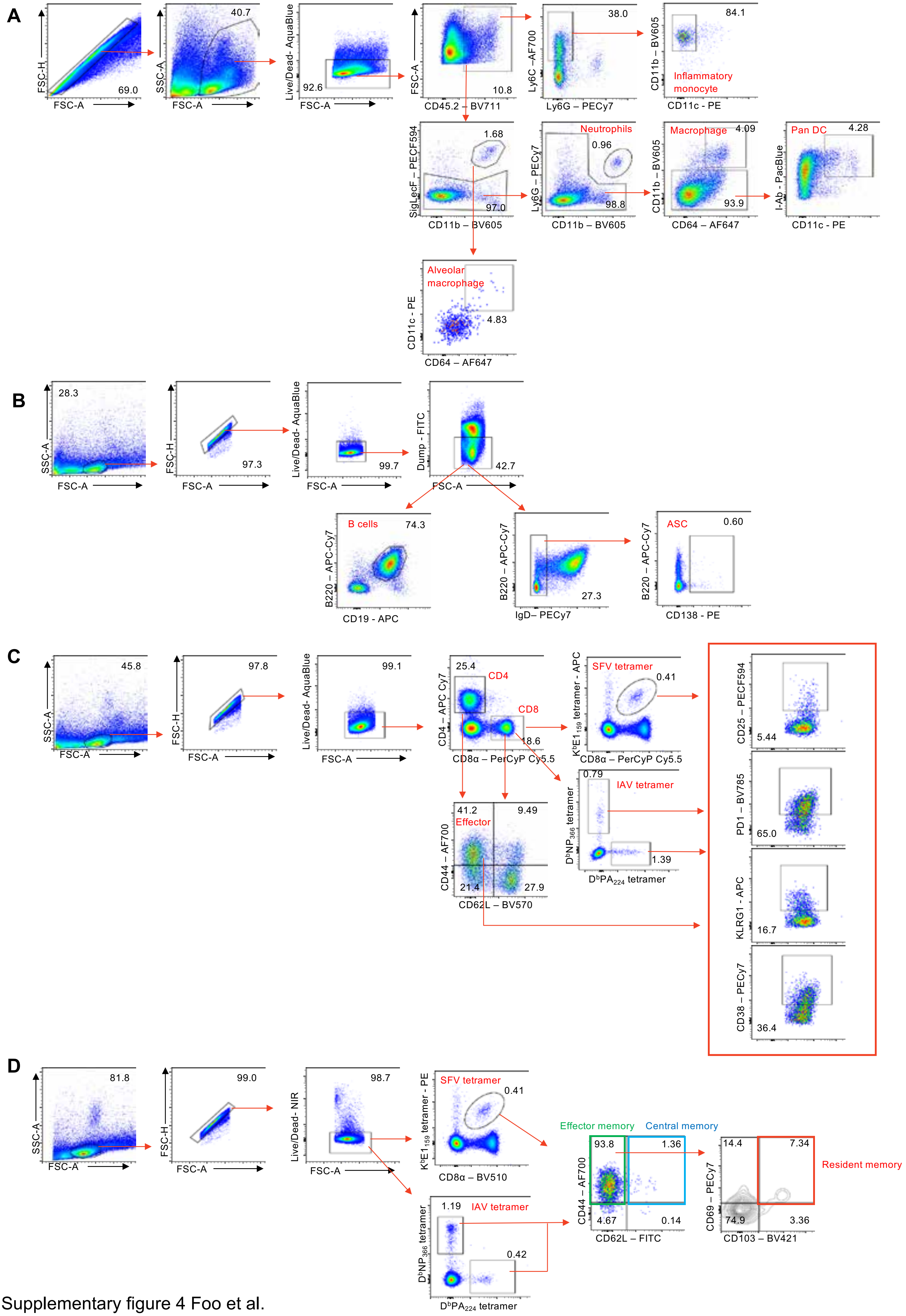

Supplementary figure 4 Foo et al.

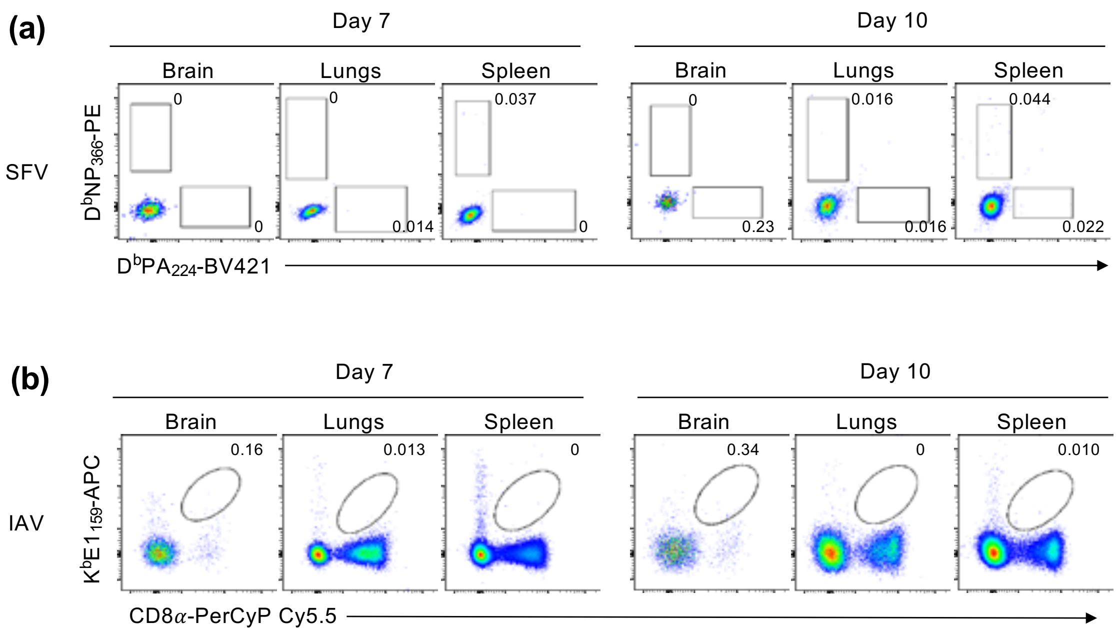

Supplementary figure 5 Foo et al.

		Percentage of damage across levels				
**Sample ID**	**Overall Grade**	**L1**	**L2**	**L3**	**L4**	**L5**
			**Brain**			
Naïve brain	GRADE 0	0%	0%	0%	0%	0%
IAV 1 D7	GRADE 0	0%	0%	0%	0%	0%
IAV 2 D7	GRADE 0	0%	0%	2%	0%	0%
IAV 3 D7	GRADE 0	0%	0%	0%	0%	0%
SFV 1 D7	GRADE 0	2%	0%	0%	0%	0%
SFV 2 D7	GRADE 1	0%	2%	5%	2%	2%
SFV 3 D7	GRADE 0	0%	0%	0%	0%	0%
SFV + IAV 1 D7	GRADE 0	0%	0%	0%	0%	0%
SFV + IAV 2 D7	GRADE 1	2%	2%	2%	2%	0%
SFV + IAV 3 D7	GRADE 0	0%	0%	0%	2%	2%
			**Lungs**			
Naïve lung	GRADE 0	0%	0%	0%	0%	0%
IAV 1 D7	GRADE 3	40%	40%	60%	60%	60%
IAV 2 D7	GRADE 3	40%	60%	60%	60%	60%
IAV 3 D7	GRADE 2	30%	40%	40%	40%	40%
SFV 1 D7	GRADE 1	15%	15%	10%	15%	15%
SFV 2 D7	GRADE 1	10%	10%	10%	10%	10%
SFV 3 D7	GRADE 1	20%	20%	10%	10%	5%
SFV + IAV 1 D7	GRADE 2	30%	30%	30%	30%	30%
SFV + IAV 2 D7	GRADE 2	40%	40%	40%	40%	40%
SFV + IAV 3 D7	GRADE 3	60%	60%	60%	60%	60%

Supplementary table 1. Histological Assessment of lung and brain during simultaneous co-infection on day 7 post-infection. Multiple sagittal sections were prepared of both hemispheres demonstrating meninges, cerebral cortex, corpus callosum, ventricles and choroid plexi, hippocampus, thalamus, midbrain and hindbrain (cerebellum, pons and medulla). Naïve: uninfected control, SFV: Semliki Forest virus infected, IAV: Influenza A virus infected, SFV + IAV: simultaneously infected with SFV and IAV. Scoring key: 0= no changes/mild changes considered insignificant; 1= minimal lesions affecting; 1-25% of the area; 2= multifocal lesions affecting 25-50% of the area; 3= severe tissue changes affecting >50% of the area.
